# Structural and Functional Evolution of the Trace Amine-Associated Receptors TAAR3, TAAR4 and TAAR5 in Primates

**DOI:** 10.1371/journal.pone.0011133

**Published:** 2010-06-15

**Authors:** Claudia Stäubert, Iris Böselt, Jens Bohnekamp, Holger Römpler, Wolfgang Enard, Torsten Schöneberg

**Affiliations:** 1 Institute of Biochemistry, Molecular Biochemistry, Medical Faculty, University of Leipzig, Leipzig, Germany; 2 Max Planck Institute for Evolutionary Anthropology, Department of Evolutionary Genetics, Leipzig, Germany; Institute of Evolutionary Biology (CSIC-UPF), Spain

## Abstract

The family of trace amine-associated receptors (TAAR) comprises 9 mammalian TAAR subtypes, with intact gene and pseudogene numbers differing considerably even between closely related species. To date the best characterized subtype is TAAR1, which activates the G_s_ protein/adenylyl cyclase pathway upon stimulation by trace amines and psychoactive substances like MDMA or LSD. Recently, chemosensory function involving recognition of volatile amines was proposed for murine TAAR3, TAAR4 and TAAR5. Humans can smell volatile amines despite carrying open reading frame (ORF) disruptions in TAAR3 and TAAR4. Therefore, we set out to study the functional and structural evolution of these genes with a special focus on primates. Functional analyses showed that ligands activating the murine TAAR3, TAAR4 and TAAR5 do not activate intact primate and mammalian orthologs, although they evolve under purifying selection and hence must be functional. We also find little evidence for positive selection that could explain the functional differences between mouse and other mammals. Our findings rather suggest that the previously identified volatile amine TAAR3–5 agonists reflect the high agonist promiscuity of TAAR, and that the ligands driving purifying selection of these TAAR in mouse and other mammals still await discovery. More generally, our study points out how analyses in an evolutionary context can help to interpret functional data generated in single species.

## Introduction

The trace amine-associated receptor family is a distinct subfamily within the family of rhodopsin-like G protein-coupled receptors (GPCR) and consists of 9 TAAR subtypes in mammals. The subtype TAAR1 was the first deorphanized TAAR and attracted attention because it is not only activated by trace amines, namely β-phenylethylamine, *p*-tyramine and tryptamine, but also by psychoactive compounds like MDMA, amphetamine and LSD [1], [2]. The signal transduction of TAAR1 is mediated by activation of the G_s_ protein/adenylyl cyclase pathway. More recently, 3-iodothyronamine, an endogenous derivative of thyroid hormones, and metabolites of the antiarrhythmic drug amiodarone were identified as agonists at TAAR1 [3], [4]. It is of interest that there are significant interspecies differences in functional and pharmacological properties of TAAR1 [5], [6], [7].

Recently, expression of several other TAAR subtypes was demonstrated in mouse olfactory epithelium. It was found that murine TAAR3, TAAR4 and TAAR5 recognize volatile amines and signal via G_s_ protein/adenylyl cyclases activation [8]. Similar to odorant receptors, TAAR show differential expansions of gene numbers among vertebrates [9], [10], [11]. Interestingly, humans do smell volatile amines that activate murine TAAR3 and TAAR4, despite the fact that these 2 genes exhibit a disrupted open reading frame in humans. Therefore we set out to study the evolution of TAAR3–5 in mammals and primates. We tested whether these TAAR are activated by the same volatile amines in different mammals and investigated their sequence evolution among primates.

We found that the agonist profiles of TAAR3, TAAR4 and TAAR5 vary significantly among mammals and that the agonists identified in mouse are unlikely to be the natural agonists that are responsible for the selective constraints observed among primates. Further, we found that pseudogenization of TAAR is common among primates and that changes in selective constraint of TAAR3 and TAAR4 are correlated, suggesting that some as yet unknown ecological factors might influence evolution of these genes. In general, the observed differences in the functional TAAR repertoire and agonist specificity may indicate species-specific or physiological TAAR functions not yet identified.

## Materials and Methods

### TAAR ortholog identification

Mining of NCBI trace archives: TAAR sequences of various mammalian species were obtained by using the respective mouse ortholog nucleotide sequences as query sequence in discontiguous megablast and blasting all available mammalian trace archives. Trace files of sequences producing significant alignments were downloaded followed by assembly, analysis (using SeqManPro of DNAStar Lasergene Software Suite for Sequence Analysis 7.1.) and manual proof-reading. One exemplary trace identifier number for each ortholog achieved is listed in Table S1.

Amplification, sequencing and cloning of TAAR orthologs: To analyze the sequence of TAAR orthologs, genomic DNA samples were prepared from tissue of various species (sources are given in Table S2). Tissue samples were digested in lysis buffer (50 mM Tris/HCl, pH 7.5, 100 mM EDTA, 100 mM NaCl, 1% SDS, 0.5 mg/ml proteinase K) and incubated at 55°C for 18 h. DNA was purified by phenol/chloroform extraction and ethanol precipitation. Degenerated primer pairs (Table S3) were applied to amplify TAAR specific sequences. PCR reactions were performed with *Taq* polymerase under variable annealing and elongation conditions. A standard PCR reaction (50 µl) contained genomic DNA (100 ng) with primers (1.5 µM each), ThermoPol reaction buffer (1x), dNTP (250 µM, each) and *Taq* polymerase (1 U, NEB, Frankfurt am Main, Germany). The reactions were initiated with a denaturation at 95°C for 1 min, followed by 35 cycles of denaturation at 95°C for 30 s, annealing at 55°C for 30 s and elongation at 72°C for 1 min. A final extension step was performed at 72°C for 10 min. Specific PCR products were directly sequenced and/or subcloned into the pCR2.1-TOPO vector (Invitrogen, Paisley, UK) for sequencing. In case of heterozygosity allelic separation was performed by subcloning and subsequent sequencing. Sequencing reactions were performed with a dye-terminator cycle sequencing kit and applied on a MegaBACE™ 1000 (GE Healthcare Europe GmbH, Munich, Germany). All obtained TAAR3–5 sequences have been deposited in the GenBank database (accession no. FJ372426–FJ372562, FJ931100–FJ931115; Table S1).

The full length TAAR were inserted into the mammalian expression vector pcDps and epitope-tagged with a N-terminal hemagglutinin (HA) epitope and a C-terminal FLAG-tag by a PCR-based overlapping fragment mutagenesis approach [12]. All TAAR4 orthologs cloned for functional testing were additionally tagged with a sequence encoding the N-terminal 20 amino acids of bovine rhodopsin N terminus as described in [8]. All TAAR4 constructs contained the 12 C-terminal amino acids (DSSTLSLFPALA) of the rhesus monkey TAAR4 as C terminus. Identity of all constructs and correctness of all PCR-derived sequences were confirmed by restriction analysis and sequencing.

### Sequence alignments and PAML analyses

Primate TAAR3 (corresponding to amino acid positions 2.58–7.70, relative numbering system of GPCR based on [13]), TAAR4 (amino acid positions 1.43–6.71) and TAAR5 (amino acid positions 1.27–7.66) nucleotide alignments were generated with the ClustalW algorithm (Bioedit Sequence Alignment Editor 7.0.9; http://www.mbio.ncsu.edu/BioEdit/bioedit.html; [14]) followed by manual trimming, whereupon frame-shifting insertions were deleted. Stop codons and triplets that were affected by 1bp or 2bp deletions in a respective ortholog were also deleted in all other sequences included in the alignment. Phylogenetic relationship of primates was inferred from our combined nucleotide TAAR3–4–5 sequence data. TAAR3–4–5 concatenation was accomplished to increase sequence input for phylogenetic tree construction. Usage of only primate TAAR3, TAAR4 and TAAR5 sequence data, respectively, did not result in fully resolved trees (see Table S4). Phylogenetic tree inference was conducted in MEGA4 [15] using the Neighbor-Joining method [16] whereas the evolutionary distances were computed applying the Maximum Composite Likelihood method [17]. In addition, we determined phylogenetic relationships from our combined TAAR3–4–5 nucleotide sequence data using Maximum Likelihood and the substitution model F84 [18] as implemented in PHYLIP3.69 [19]. Branch support was estimated with 1,000 bootstrap replicates [20]. The resulting trees (Figure S1A, B) are not fully resolved indicated by bootstrap values below 95%, but do not contradict other reconstructions of primate phylogeny that use much more sequence data [21]. Consequently, for PAML analyses we assumed a primate phylogeny (Table S4) determined by [21]. Pseudogenes were removed from the respective trees for performance of PAML analyses of primate TAAR3–5 ORF lineages. Tests of selection (ω = d_N_/d_S_) were accomplished by maximum likelihood using a codon-based substitution model implemented in PAML version 4.2 [22]. Branch models [23] that allow ω to vary among branches in the phylogeny were applied to determine ω ratios on particular lineages. Different site models [24] that allow ω to vary between sites were tested (models M1a, M2a, M3, M7, M8). Comparison of 2 pairs of models was performed: M1a and M2a, M7 and M8. Branch-site models were applied to detect positive selection affecting only a few sites on pre-specified foreground branches (test 2: modified model A versus modified model A with ω_2_ = 1 fixed) [25], [26]. All analyses were run twice using different initial ω values to check for convergence. Likelihood ratio tests (LRT) were performed to test nested competing hypotheses.

Orthologous TAAR3, TAAR4 and TAAR5 full length nucleotide sequences of 14 mammalian species (Table S1 all species included are marked with *) were imported into Bioedit Sequence Alignment Editor 7.0.9 (http://www.mbio.ncsu.edu/BioEdit/bioedit.html) and aligned using ClustalW [14] followed by manual trimming as stated above. Inference of phylogenetic relationship of these 14 mammals from concatenated TAAR3–4–5 nucleotide sequence data using Neighbour-Joining and Maximum Likelihood methods (see above for details) did not result in fully resolved trees (Figure S2A, B). Hence, for PAML analyses phylogenetic relations of these 14 mammals determined by [27] was assumed. Different branch models [22], site models [24] and branch-site models implemented in PAML version 4.2 were applied [25], [26] as described above.

### Estimates of inactivation time and chances

The rate of inactivating mutations of TAAR sequences under neutral evolution was determined by counting all pseudogenization events in branches in which the respective TAAR is already inactivated by deleterious mutations (frame-shifting indels, stop codons) in its ancestral branch assuming the primate phylogeny as stated above. Rates were calculated as events per unit of time using divergence times of primate species as given in [28], [29] and as events per unit of d_S_ distance. ReEVOLVER 1.0 was used to determine the probability (*P_dis_*) that the observed number of deleterious mutations (stop codons, frame-shifting indels) is lower or equal to what would be expected under neutral evolution [30]. *P_dis_* is computed through comparison of the observed value with the frequency distribution generated by simulation of neutral evolution. An indel rate of 1.0×10^−10^ per site per year and a mutation rate of 1.0×10^−9^ per site per year as suggested for Old World monkeys [30] was used and 10,000 simulations were performed.

### Correlation of ω in TAAR paralogs

A phylogenetic tree was constructed containing only primate species for which sequences of all 3 receptors, TAAR3, TAAR4 and TAAR5, were available (Table S4). The “free ratio” model implemented in PAML was used to calculate a separate ω value for each branch for each receptor (Table S4). A Spearman rank correlation was performed on ω values using GraphPad Prism version 5.01 to pairwise evaluate association of evolution between TAAR3, TAAR4 and TAAR5. Branches which showed d_S_ = 0 (absence of synonymous changes, leading to odd ω values meaning infinity) or branches which showed d_N_ = 0 (absence of non-synonymous changes, meaning that ω values approximate 0) were excluded from analyses.

### Cell culture and functional assays

HEK293 cells were grown in Minimum Essential Medium (MEM) supplemented with 10% fetal bovine serum, 100 U/ml penicillin, and 100 µg/ml streptomycin at 37°C in a humidified 7% CO_2_ incubator. One day prior to transfection cells were split into 50-ml cell culture flasks (1.4x10^6^ cells/flask) and, for the ALPHAScreen™ cAMP assay, transfected with a total amount of 4 µg plasmid. For the CRE-SEAP (secreted alkaline phosphatase) reporter gene assay cells were co-transfected (3 µg of each) with the TAAR expression plasmid and the CRE-SEAP reporter plasmid (Clontech, Saint-Germain-en-Laye, France). Lipofectamine™ 2000 (Invitrogen, Paisley, UK) was used for transient transfection of HEK293 cells.

ALPHAScreen™ cAMP assay: cAMP content of cell extracts was determined by a non-radioactive cAMP accumulation assay based on the ALPHAScreen™ technology according to the manufacturers' protocol (Perkin Elmer LAS, Rodgau-Jügesheim, Germany) [31]. One day after transfection cells were split into 48-well plates (8x10^4^ cells/well). Stimulation with various agonist concentrations (all compounds from Sigma-Aldrich, Seelze, Germany) was performed 48 h after transfection. Reactions were stopped by aspiration of media and cells were lysed in 50 µl of lysis buffer containing 1 mM 3-isobutyl-1-methylxanthine. From each well 5 µl of lysate were transferred to a 384-well plate. Acceptor beads (in stimulation buffer without 3-isobutyl-1-methylxanthine) and donor beads were added according to the manufacturers' protocol.

CRE-SEAP-reporter gene assay: One day after transfection HEK293 cells were split into 96-well plates (4x10^4^ cells/well) and serum-free medium with no and increasing concentrations of compounds was added the following day. Cells were incubated for 24 h at 37°C and then for 2 h at 65–70°C. An aliquot of the supernatant from each well was then incubated (2–5 min, 21°C) with an equal volume of 1.2 mM 4-methylumbelliferyl phosphate (Sigma-Aldrich, Seelze, Germany) in 2 M diethanolamine bicarbonate with 1 mM MgCl_2_ and 4.5 mg/ml L-homoarginine (pH 10) and fluorescence was measured with a Victor 2–1420 Multilabel counter (Perkin Elmer LAS, Rodgau-Jügesheim, Germany).

Both cyclic AMP accumulation data and CRE-SEAP-reporter gene assay data were analyzed using GraphPad Prism version 5.01 for Windows (GraphPad Software, San Diego California USA, www.graphpad.com).

## Results

### Evolution of open reading frame disruptions in TAAR3, TAAR4 and TAAR5

**Figure 1:**
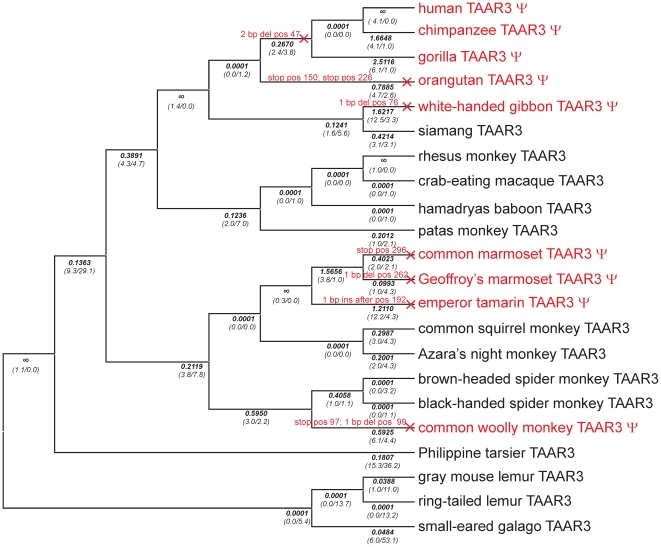
Phylogenetic tree of primate TAAR3 orthologs analyzed for open reading frames in the present study. The tree is based on generally accepted primate phylogeny as described in [21]. Events causing pseudogenization (nucleotide insertions (ins) or -deletions (del) or stop mutations (stop)) were determined and are labeled on the affected branches in red. Positions stated correspond to codon position of the respective mouse ortholog. Pseudogenes are indicated as ψ and highlighted in red. Detailed information about the sequence changes causing pseudogenization are in Figure S3. d_N_/d_S_-ratios (ω) for each branch were calculated by using a “free ratio” model implemented in PAML and are shown in bold below the branches. The number of non-synonymous and synonymous substitutions is given in parentheses. × indicates branches that were labeled to determine ω_ψ2_ (see Table 1, Table S6).

**Figure 2 pone-0011133-g002:**
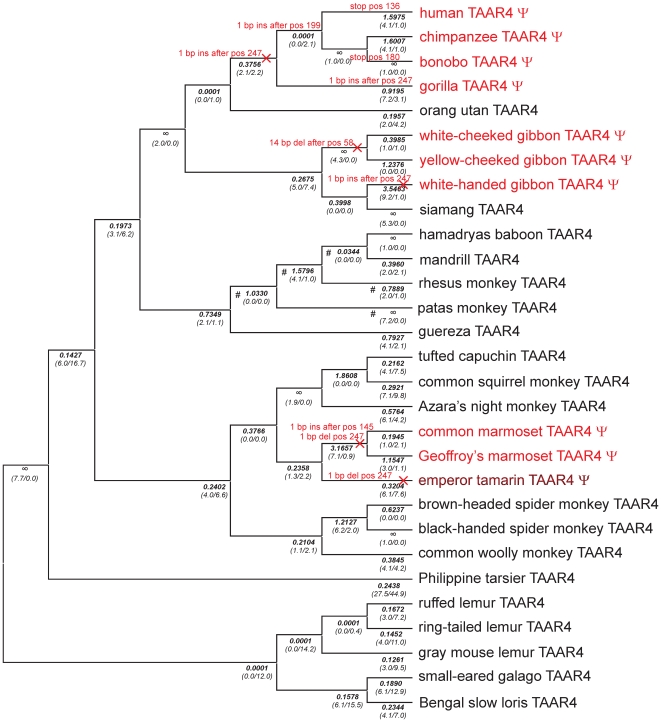
Phylogenetic tree of all primate TAAR4 orthologs analyzed for ORF. The tree is based on generally accepted primate phylogeny as described in [21]. Events causing pseudogenization (nucleotide insertions (ins) or -deletions (del) or stop mutations (stop)) are indicated in red on the affected branches. Positions stated correspond to codon position of the respective mouse ortholog. Pseudogenes (ψ) are highlighted in red. Emperor tamarin was found to be polymorphic. Detailed information about pseudogenization events is in Figure S4. A “free ratio” model implemented in PAML was used to calculate d_N_/d_S_-ratios (ω shown in bold below branches) and the number of non-synonymous and synonymous substitutions (shown in parentheses) for each branch. × indicates branches that were labeled to determine ω_ψ2_ (see Table 1, Table S6) # indicates branches labeled in branch model/branch-site model to determine ω_cerc_.

**Figure 3:**
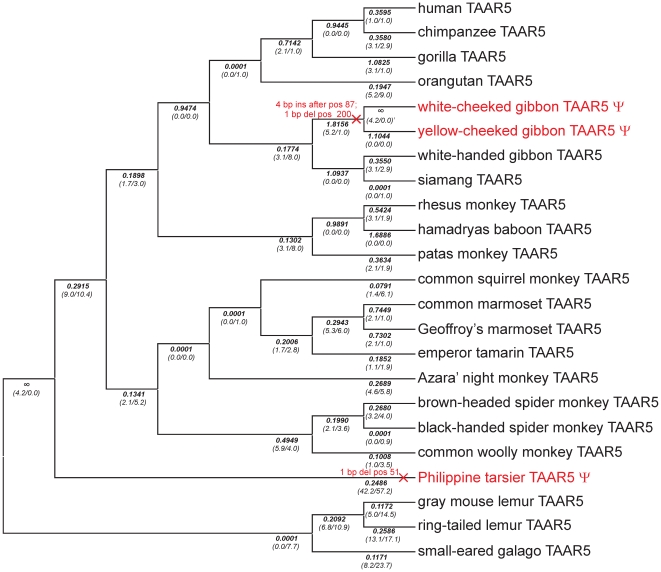
Phylogenetic tree of primate TAAR5 orthologs analyzed for ORF. The tree is based on generally accepted primate phylogeny as described in [21]. ORF disruptions (ψ highlighted in red) are only found in Philippine tarsier (1bp deletion) and white- and yellow-cheeked gibbon TAAR5 (4bp insertion and 1bp deletion). Positions stated correspond to codon position of the respective mouse ortholog. Detailed information about pseudogenization events is depicted in Figure S5. Below the branches, d_N_/d_S_-ratios (ω) that were determined by using a “free ratio” model implemented in PAML are shown. The number of non-synonymous and synonymous substitutions is indicated in parentheses. × indicates branches that were labeled to determine ω_ψ2_ (see Table 1, Table S6).

Full length or partial TAAR3, 4 and 5 sequences of altogether 103 species were obtained by cloning and sequencing various mammalian orthologs in particular from primates, and by mining public sequence databases for additional orthologous sequences (Table S1). To map mutational events we used the primate phylogeny as stated in [21]. All 3 genes show mutational events on different lineages that disrupt the ORF (Figures 1, 2 and 3). Disruptions of TAAR3 ORF are caused by 7 independent events, 3 in apes and 4 in New World monkeys, 3 of which occurred in the family *Callithrichinae* (Figure 1, Figure S3). A similar pattern is seen for the 4 independent disruptions of TAAR4 (Figure 2, Figure S4). ORF disruptions in TAAR5 occurred only in the gibbon lineage and the lineage leading to the tarsier (Figure 3, Figure S5). In contrast to primates, only very few ORF disruptions were detected in other mammals analyzed. We found that 1 out of 25 non-primate species carry a TAAR3 or a TAAR5 pseudogene. For TAAR4 100 species (31 *Primates*, 11 *Glires*, 1 *Scandentia*, 21 *Carnivora*, 4 *Perissodactyla*, 11 *Cetartiodactyla*, 5 *Chiroptera*, 4 *Insectivora*, 3 *Xenarthra*, 6 *Afrotheria*, 2 *Metatheria*, 1 *Protheria*) were analyzed. We detected 10 TAAR4 pseudogenes (out of 31) in primates, but only 1 (out of 21) in *Carnivora*. Of 6 *Afrotheria*, only Caribbean manatee and lesser hedgehog tenrec carry a TAAR4 pseudogene whereas the latter possess an additional intact copy of TAAR4. Western European hedgehog and European shrew exhibit also both 1 intact and 1 pseudogene TAAR4 copy. In sum, we observed ORF disruptions also in other mammals, but significantly less frequent than in primates (Table S1).

### Functional characterization of selected mammalian TAAR3, TAAR4 and TAAR5 orthologs

**Figure 4:**
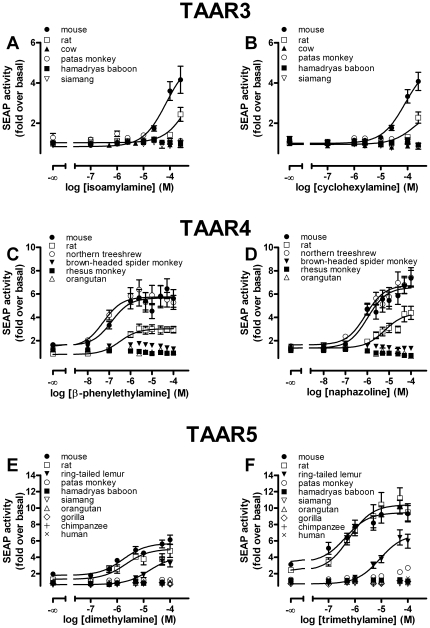
Functional characterization of mammalian TAAR3, TAAR4 and TAAR5 orthologs using a CRE-SEAP reporter gene assay. HEK293 cells were transiently co-transfected with CRE-SEAP reporter plasmid (Clontech) and respective receptor orthologs and tested for agonist-induced SEAP-activity. The basal value of non-stimulated mock-transfected HEK293 determined was 193,208±21,052 cpm/well. Data are given as mean±SEM of 2 to 5 independent experiments each performed in triplicates. Concentration-response curves of agonists were determined using GraphPad Prism.

Since agonists for mouse TAAR3–5 have been identified [8], we next asked whether these agonists also activate intact orthologs of other mammals. In addition to the mouse TAAR3–5, which served as positive control, we included the rat TAAR3–5 as orthologs from another rodent species, selected primate TAAR3–5 containing intact ORF and at least one ortholog from a non-primate/non-rodent species like cow, dog or northern treeshrew in functional analyses. It was previously shown using a CRE-SEAP reporter gene assay that isoamylamine and cyclohexylamine stimulate intracellular cAMP formation via activation of the mouse TAAR3 [8]. Whereas we found activation of mouse TAAR3 by these agonists in a CRE-SEAP reporter gene assay, none of the tested intact primate TAAR3 orthologs (hamadryas baboon, rhesus monkey, emperor tamarin) displayed activity (Figures 4A, B). Furthermore, the bovine TAAR3 did not show any detectable activity and even the rat TAAR3, which is 95.6% identical to the mouse TAAR3 protein, exhibited much lower efficacy and potency to both agonists (Figures 4A, B).

**Figure 5:**
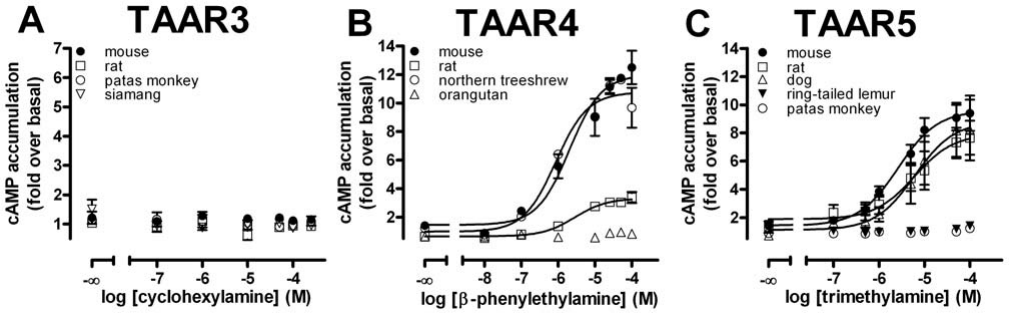
Functional characterization of mammalian TAAR3, TAAR4 and TAAR5 orthologs using a cAMP accumulation assay. Cells were transfected with receptor orthologs and agonist-induced cAMP accumulation was determined with the ALPHAScreen™ technology. (*see Material and Methods*). The basal cyclic AMP level of non-stimulated mock-transfected HEK293 was 6.35±1.02 amol/cell. Data are given as mean±SEM of 2 independent experiments each performed in duplicates. Concentration-response curves of agonists were determined using GraphPad Prism.

It has been shown that agonistic properties of ligands can differ considerably between the CRE-SEAP reporter gene assay and classical cAMP accumulation assays [32]. Therefore, TAAR3 orthologs were additionally tested in an ALPHAScreen™ proximity assay where accumulated cAMP is measured directly. Surprisingly, neither the murine TAAR3 nor the other TAAR3 orthologs tested displayed cAMP production following incubation with cyclohexylamine (Figure 5A) and isoamylamine (data not shown) although the assay worked properly with TAAR4 and TAAR5 (Figures 5B, C).

It was demonstrated that the murine TAAR4 couples to the G_s_ protein/adenylyl cyclase pathway and is activated by β-phenylethylamine when N-terminally tagged with 20 amino acids of the bovine rhodopsin N terminus [1], [8]. Consistent with this, we found that β-phenylethylamine acts as agonist at the mouse, rat and northern treeshrew TAAR4 orthologs in both the CRE-SEAP reporter gene assay and the cAMP accumulation assay (Figures 4C, 5B). However, no activation by β-phenylethylamine was observed for the 3 tested intact primate TAAR4 orthologs (Figure 4C).

Di- and trimethylamine act as agonists at mouse, rat (Figures 4E, F) and dog TAAR5 (data not shown). Nonetheless, functional analyses of selected primate TAAR5 orthologs with intact ORF revealed that only the ring-tailed lemur and patas monkey TAAR5 orthologs can be activated by di- and trimethylamine to a marginal extent (Figures 4E, F). Both methylamines displayed no agonistic activity at all other tested primate (Figures 4E, F) and cow TAAR5 (data not shown) in the CRE-SEAP reporter gene assay. In cAMP accumulation assays di- and trimethylamine were agonists at the mouse, rat and dog TAAR5, but no activation was found for the ring-tailed lemur and patas monkey orthologs (Figure 5C).

In summary, we found that the agonists identified for mouse TAAR3–5 are generally not agonists at primate TAAR3–5. To gain further insight into the forces that might influence the evolution of TAAR3–5 in primates we analyzed their patterns of sequence evolution.

### Sequence evolution of TAAR3, TAAR4 and TAAR5 in primates

Although the structural conservation of intact TAAR on the amino acid level is similar to other GPCR (Table S5) our functional analyses revealed that agonist specificity for volatile amines is much less conserved. This can be explained by different scenarios. A *first* possibility is that even obviously intact TAAR contain inactivating missense mutations, but have just by chance not acquired ORF disruptions, yet. A *second* possibility is that changes in agonist specificity were advantageous during evolution, i.e. that positive selection acted on some TAAR to recognize ligands other than the known mouse agonists. A *third* possibility is that intact TAAR are activated by a yet unknown endogenous agonist and the volatile amines are promiscuous agonists, identified by chance. Evidence for and against these hypotheses can be gathered by examining the relative rate of amino acid fixations, so we estimated d_N_/d_S_ ratios (ω) across the different primate lineages.

The d_N_/d_S_ ratio is the number of non-synonymous substitutions per non-synonymous site (non-synonymous substitution rate d_N_) normalized to the number of synonymous substitutions per synonymous site (synonymous substitution rate d_S_). If a protein evolves under no constraint, i.e. is a pseudogene and has no function, it is expected to show an ω of 1, since synonymous mutations get fixed as frequently as non-synonymous mutations. Most proteins show an ω well below 1, since most mutations that lead to amino acid substitutions decrease fitness and hence are unlikely to become fixed between species (purifying selection). In contrast, amino acid substitutions that are positively selected are more likely to become fixed and repeated positive selection can be detected by an ω significantly larger than 1. We used PAML to estimate ω and compare different codon substitution models in a maximum-likelihood framework [22], [33]. Note however, that absence of evidence for positive selection in PAML does not rule out presence of positive selection since e.g. positive selection acting rarely on one or a few sites is unlikely to be detected [34].

To investigate the *first* scenario, we tested whether intact primate TAAR3–5 ORF evolve under purifying selection by comparing different models (Table 1, Table S6). We found that a model in which lineages that contain ORF disruptions have a different ω to all other lineages is strongly favored for TAAR3 and TAAR4 (model B versus model C, Table 1, Table S6). In the case of TAAR5 there are just 2 lineages with ORF disruptions whereas one is the long branch leading to the Philippine tarsier. This lineage has a rate significantly smaller than 1 (model D versus model C, Table 1, Table S6). This can be readily explained if the loss of constraint and the subsequent ORF disruption occurred a substantial time after the divergence of the lineage, since in this case ω calculated for the lineage is a mixture of earlier evolution under constraint and later neutral evolution. Therefore, we distinguished branches that had an ORF disruption acquired already in an ancestral branch (ψ_1_ lineages) from lineages along which a pseudogenization event occurred (ψ_2_ lineages, see Figures 1, 2 and 3). In such a model the ψ_1_ lineages indeed evolve at a rate that is not different from 1 as expected (model G versus model F, Table 1, Table S6). Lineages with intact ORF evolve at rates significantly (p<0.0001) smaller than 1 (model E versus model C, Table 1, Table S6) and we estimate these rates to be 0.128, 0.274 and 0.213 for TAAR3, TAAR4 and TAAR5, respectively (model C, Table S6). Taken together, these analyses clearly show that TAAR pseudogenes evolve without constraint and TAAR with intact ORF evolve under constraint, making it very unlikely that the observed functional differences result from intact but non-functional TAAR in primates. However, for individual branches it is in principle still possible that the loss of constraint occurred very recently, and that while missense mutations led to a change or loss of functionality as demonstrated in other GPCR [35], ORF disruptions have by chance not yet occurred. Therefore, we estimated the average time for acquiring at least one ORF disrupting mutation in TAAR3–5 under neutral evolution. The decisive parameter hereby is the rate of frame-shift mutations since these are more likely to occur than stop mutations [36]. Frame-shift mutations depend strongly on sequence context, as can be seen in 4 independent insertion and deletion events in the TAAR4 poly-A stretch at codon position 247–249 (Figure 2, Figure S4). We used the empirical rate of pseudogenization events that occurred in all lineages of TAAR3–5 that had already acquired an ORF disruption in an ancestral lineage. We estimate that an ORF disruption occurs once every 7.3±2.7 million years (myr) (assuming a Poisson distribution) or once per 2.2±1.7 synonymous substitutions. Hence, it is not very likely but in principle possible that some of the tested intact primate TAAR are non-functional due to a recent loss of constraint and inactivating missense mutations. However, it is extremely unlikely that all tested intact primate TAAR are not activated by the mouse agonists for this reason. A far more parsimonious explanation is certainly that most if not all intact TAAR are functional since they evolve under strong constraints and would acquire ORF disruptions relatively quickly after losing this constraint.

**Table 1:** Likelihood ratio test (LRT) statistics comparing different models presented in Table S6 for analyses of pseudogene evolution of TAAR3–5.

Hypothesis tested	TAAR3		TAAR4		TAAR5	
(models compared: null vs. alternative)^a^	ω	*P*-value	ω	*P*-value	ω	*P*-value
ω_0_≠ω_ψ_	ω_0_ = 0.128	<0.0001	ω_0_ = 0.274	<0.0001	ω_0_ = 0.213	0.1198
(B vs. C)	ω_ψ_ = 0.878		ω_ψ_ = 0.884		ω_ψ_ = 0.319	
ω_0,_ ω_ψ_≠1	ω_0_ = 0.128	0.5967	ω_0_ = 0.274	0.6390	ω_0_ = 0.213	<0.0001
(D vs. C)	ω_ψ_ = 1		ω_ψ_ = 1		ω_ψ_ = 1	
ω_0_≠1, ω_ψ_	ω_0_ = 1	<0.0001	ω_0_ = 1	<0.0001	ω_0_ = 1	<0.0001
(E vs. C)	ω_ψ_ = 0.920		ω_ψ_ = 0.903		ω_ψ_ = 0.323	
ω_0_≠ω_ψ_	ω_0_ = 0.128	0.0444	ω_0_ = 0.274	0.6714	ω_0_ = 0.213	0.0119
ω_ψ_≠ω_ψ1_≠ω_ψ2_	ω_ψ1_ = 2.915		ω_ψ1_ = 0.787		ω_ψ1_ = ∞	
(F vs. C)	ω_ψ2_ = 0.701		ω_ψ2_ = 0.982		ω_ψ2_ = 0.285	
ω_0_, ω_ψ1_≠1, ω_ψ2_	ω_0_ = 0.128	0.1089	ω_0_ = 0.274	0.5271	ω_0_ = 0.213	0.1153
(G vs. F)	ω_ψ1_ = 1		ω_ψ1_ = 1		ω_ψ1_ = 1	
	ω_ψ2_ = 0.699		ω_ψ2_ = 0.982		ω_ψ2_ = 0.285	
ω_0_, ω_ψ1_, ω_ψ2_≠1	ω_0_ = 0.129	0.1871	ω_0_ = 0.274	1	ω_0_ = 0.213	<0.0001
(H vs. F)	ω_ψ1_ = 2.924		ω_ψ1_ = 0.787		ω_ψ1_ = ∞	
	ω_ψ2_ = 1		ω_ψ2_ = 1		ω_ψ2_ = 1	

a LRT tests were performed between nested models. In parentheses designation for the compared models is given as indicated in Table S6. ω, d_N_/d_S_ ratio; ω_0_, indicates ω of all other branches (the ones that are not specifically labeled in a model); ω_ψ1_, ω of branches being definitely under neutral evolution because of deleterious mutation (pseudogenization) in ancestor branch; ω_ψ2_, ω of branches along which inactivating pseudogenization event occurred (see × Figure 1–3); ω_ψ_, ω of all pseudogene branches (_ψ1_ plus _ψ2_); ∞, ω estimated to be infinite generated by d_S_ = 0 (absence of synonymous changes).

The *second* scenario was examined by exploring evidence for positive selection. We first tested whether models in which some sites in intact TAAR evolve at rates larger than 1 explain the sequence differences significantly better than models without such sites (site models Table S6, Table S7). We found no evidence for positively selected sites in primate TAAR3–5. Although, this does not exclude that some positive selection occurred during primate TAAR evolution, it argues against frequent positive selection acting on the same sites within primates and therefore against positive selection as a cause for agonist change within primates. However, we found evidence for positively selected sites among 14 mammalian non-primate TAAR5, but not among non-primate TAAR3 and TAAR4 (Table S6, Table S7). Moreover, we applied the “free ratio” model implemented in PAML to determine ω values for each branch for each receptor for primates and mammals (Figures 1, 2 and 3, Figure S7). Since multiple lineages were evaluated this way, multiple testing is a potential problem for inferring the significance of results. However, the analysis is still useful for exploring branches especially if functional assays are available to support claims about positive selection by additional experiments. For TAAR3 of primates and other mammals there are no lineages evolving at a suspiciously high rate. Thus, we find no evidence at all that positive selection changed the agonist profile of TAAR3. Moreover, non-function of cow TAAR3 and primate TAAR3 argue against a single amino acid substitution as a cause for the functional differences observed. Together with the marginal and no activity of mouse and rat TAAR3 in the CRE-SEAP reporter gene assay and the cAMP accumulation assay, respectively, this suggests that the identified agonistic volatile amines at TAAR3 are not the natural agonists.

For TAAR4, lineages of *Cercopithecinae* (rhesus monkey, mandrill, hamadryas baboon and patas monkey) and *Glires* (rabbit, guinea pig, rat and mouse) showed a high ω. Application of a branch model in which TAAR4 of *Cercopithecinae* were labeled as foreground branch (see Figure 2) revealed a significantly higher ω (ω_cerc_ = 2.590) compared to all other branches (Table 2). However, TAAR4 in *Cercopithecinae* did not evolve at a rate significantly larger than 1 and also showed no explicit evidence for specific sites under positive selection (branch-site model, Table 2, Table S6). Because higher ω in *Cercopithecinae* can be taken as a signal of neutral evolution we simulated neutral evolution using ReEVOLVER 1.0 [30] and found that it is very unlikely to observe no disrupting mutations in *Cercopithecinae* TAAR4 orthologs (*P*_dis_ = 0.0006) within 55 myr of neutral evolution (or 7.2 synonymous substitutions, respectively). Similarly, TAAR4 of *Glires* evolved with a significantly higher ω (ω_glires_ = 1.209) than all other mammalian branches but not significantly different from 1 (Table 2). Using branch-site model A with the *Glires* branch (see Figure S7B for labeling) as foreground lineage in the branch-site test of positive selection (null model ω_2_ = 1 fixed) LRT provided marginally significant support (*P* = 0.052) for 2 positively selected sites with Bayes empirical Bayes (BEB) probability above 0.7 (Table 2). Therefore, there is some evidence that positive selection might be responsible for different agonist profiles found for TAAR4. Future attempts of deorphanization of TAAR4 in primates and other mammals should take into account that TAAR4 of some tailed Old World monkeys and rodents might have a different function.

**Table 2:** Likelihood ratio test (LRT) statistics for testing hypotheses (models shown in Table S6) concerning positive selection in certain species of TAAR4 and changing constraint in TAAR5.

Hypothesis tested	ω	*P*-value
**TAAR4**		
*branch models*		
ω_0_≠ω_cerc_	ω_0_ = 0.247	<0.0001
	ω_cerc_ = 2.590	
ω_0,_ ω_cerc_≠1	ω_0_ = 0.247	0.1626
	ω_cerc_ = 1	
ω_0_≠ω_glires_	ω_0_ = 0.172	0.0477
	ω_glires_ = 1.209	
ω_0,_ ω_glires_≠1	ω_0_ = 0.172	0.8875
	ω_glires_ = 1	
*branch site models*		
model A versus model A'	ω_0_ = 0.153	0.1389
(foreground branch: cerc)	ω_1_ = 1	
	ω_2(cerc)_ = 3.722	
	ω_2a(othprim)_ = 0.153	
	ω_2b(othprim)_ = 1	
model A versus model A'	ω_0_ = 0.079	0.0525
(foreground branch: *Glires*)	ω_1_ = 1	
	ω_2(glires)_ = 42.11	
	ω_2a(othmam)_ = 0.079	
	ω_2b(othmam)_ = 1	
**TAAR5**		
*branch models*		
ω_0_≠ω_hcg_	ω_0_ = 0.204	0.0676
	ω_hcg_ = 0.537	
ω_0,_ ω_hcg_≠1	ω_0_ = 0.205	0.2542
	ω_hcg_ = 1	
ω_0_≠ω_rp_	ω_0_ = 0.213	0.2965
	ω_rp_ = 0.543	
ω_0,_ ω_rp_≠1	ω_0_ = 0.213	0.5169
	ω_rp_ = 1	

ω_cerc_, ω of *Cercopithecinae* (see Figure 2 for labeling); ω_glires_, ω of *Glires* (see Figure S7B for labeling); ω_hcg_, ω of human, chimp and gorilla; ω_rp_, ω of rhesus monkey and hamadryas baboon; ω_othprim_, ω of all other primates except species labeled as foreground branches in respective branch-site test of positive selection; ω_othmam_, ω of all other mammals except species labeled as foreground branches in branch-site test of positive selection.

Finally, application of a “free ratio” model to TAAR5 of non-primate mammals did not reveal signs of positive selection in any branches. The same approach applied to primate TAAR5 with intact ORF revealed a few branches with increased ω values (especially within apes and at the split of rhesus monkey and hamadryas baboon). However, LRT failed to provide significant support for positive selection along these branches (see Table 2) and it is more parsimonious to assume that positive selection has not changed the agonist profile of TAAR5 especially because no function was observed in TAAR5 of different mammalian orders (*Primates*, *Cetartiodactyla*). Overall, evolutionary and functional data imply that agonist profiles changed frequently in TAAR5 and that the volatile amines are species-specific surrogate but not the natural agonists.

In sum, phylogenetic sequence analyses neither strongly support the *first* nor the *second* scenario as explanations for the fact that primate TAAR are not activated by the respective mouse agonists. This leaves the *third* scenario as most likely explanation which we further investigated by searching compound libraries for additional agonists at murine TAAR3–5 using CRE-SEAP reporter gene assays. We identified naphazoline (see Figure 4D), xylometazoline and β-methylphenylethylamine (see Figure S6) as agonists at mouse TAAR4. These substances also activated rat and northern treeshrew, but not primate TAAR4 (data not shown). These findings clearly demonstrate agonist promiscuity at least for TAAR4 which has also been shown for TAAR1 by previously published functional data [5], [6], [7]. It is therefore reasonable to assume that volatile amines act as promiscuous agonists at some TAAR3 and TAAR5 orthologs, but are not the endogenous agonists constraining evolution on these receptors.

### Test for correlated evolution of TAAR3–5 in primates

Sequence analyses of primate TAAR3–5 for pseudogenization events (see above) revealed an apparent overlap among species (in apes and *Callithrichinae*) that carry TAAR3 and TAAR4 pseudogenes (Figures 1, 2). We therefore explored to what extent the rate of evolution is correlated among TAAR3–5 at the different primate branches. Thus, lineages were grouped into 4 categories, in which pseudogenization occurred in both TAAR3 and TAAR4 (category 1) or only in TAAR3 (category 2) or TAAR4 (category 3), or in neither gene (category 4). We only included species in this test for which sequence information is available for all 3 analyzed subtypes (see Figure S1) and found 3 events in category 1, 4 in category 2, 1 in category 3 and 31 in category 4. We detected a significant overlap of pseudogene lineages between TAAR3 and TAAR4 (Fisher's exact test, *P* = 0.01) whereas TAAR3 and TAAR5 or TAAR4 and TAAR5 share no overlap (*P* = 1). To further investigate the potentially correlated evolution of TAAR3 and TAAR4, we correlated ω values for each branch generated by “free ratio” models in PAML (Table S4) to pairwise evaluate correlation of TAAR evolution. We found a significant correlation of d_N_/d_S_ ratios of TAAR3 and TAAR4 (Spearmans' rank r_s_ = 0.4870, *P* = 0.0252) whereas the 2 other combinations showed no correlation (TAAR3–TAAR5, coefficient r_s_ = 0.3345, *P* = 0.1383; TAAR4–TAAR5, coefficient r_s_ = 0.3754, *P* = 0.0935). This indicates that selective pressures that differ among species affect the amount of constraint acting on TAAR3 and TAAR4 in a similar manner and thus suggest a similar function of these 2 receptors.

## Discussion

### Mouse TAAR3, TAAR4 and TAAR5 agonists do not account for purifying selection on intact primate receptors

Recently, several volatile amines were found to activate several murine TAAR subtypes including TAAR3 (isoamylamine, cyclohexylamine), TAAR4 (β-phenylethylamine) and TAAR5 (di- and trimethylamine) [8]. These findings were intriguing in assigning TAAR as an important receptor class of the chemosensory system. However, all humans carry only pseudogenes of TAAR3 and TAAR4 but are nevertheless able to smell these amines, questioning the generality of the findings for mouse TAAR.

Our studies confirmed the previously identified agonists at murine TAAR3, TAAR4 and TAAR5 in the CRE-SEAP reporter gene assays (see Figure 4). In contrast, most intact primate TAAR3–5 showed no activation upon stimulation with the respective amines. Only the ring-tailed lemur TAAR5 displayed marginal activity when incubated with di- and trimethylamine (see Figures 4E, F). A loss of agonist potency and/or efficacy is already seen when mouse and rat TAAR3 and TAAR4 orthologs are compared (see Figures 4A–D) indicating species-specific agonist specificity. In case of TAAR4, this pharmacological difference is caused by only a few amino acid differences (see Figure S8). The CRE-SEAP reporter gene assay used by [8] is convenient but cAMP formation is read out indirectly and downstream the signaling cascade. Therefore, we tested whether the differences in ortholog function are also present when cAMP formation upon stimulation was measured directly in a cAMP accumulation assay. Surprisingly, receptor activation was found for rat and mouse TAAR4 and TAAR5 but not for any TAAR3 ortholog (see Figures 5A–C). We speculate that the sensitivity of the CRE-SEAP reporter gene assay is higher than that of the classical cAMP accumulation assay. It has been demonstrated that CREB can be activated also by other signal transduction pathways and results from the CRE-SEAP reporter gene assay can differ when compared with other second messenger assays [32], [37]. Nevertheless, the fact that almost all primate and other intact mammalian TAAR3–5 orthologs lacked activity upon agonist stimulation in both assays implicates that the volatile amine agonists at the murine TAAR are not the agonists for most other mammal and primate orthologs.

We used phylogenetic sequence analyses to evaluate different scenarios as explanation for these observed functional differences. One possibility is that the tested mammalian and primate genes are non-functional due to missense mutations despite their intact ORF. However, the low ω observed among primates and mammals in general (see Table S6) together with the high probability of obtaining ORF disruptions after a release of constraint makes this scenario very unlikely. Another possibility is that the physiological function of these TAAR may have changed frequently during evolution. This is unlikely for TAAR3, because we did not find any signs of positive selection. These results together with the discrepancies in functional data obtained on TAAR3 in different assays instead suggest that the previously identified agonists are surrogate ligands but not the natural agonists that drive purifying selection on this gene in mouse and other mammals. For TAAR4 there are some indications for positive selection in some tailed Old World monkeys and in *Glires* (including rat and mouse). Thus, it is possible that the recognition of the formerly identified agonist β-phenylethylamine is functionally relevant for *Glires*, but the agonist activating most other mammalian TAAR4 still needs to be identified.

The hypothesis that the identified volatile amines are surrogate agonists at TAAR3–5 is in line with the high ligand promiscuity of TAAR1 [5], [6], [7]. In addition to trace amines (β-phenylethylamine, *p*-tyramine, octopamine, tryptamine), other biologically active compounds are potent and efficient TAAR1 agonists including amphetamines and thyronamines among the most notable [38]. Similarly, we found naphazoline (Figure 4F), xylometazoline and β-methylphenylethylamine (Figure S6), to be full or partial agonists on several TAAR4 orthologs whereas many TAAR1 agonists (*p*-tyramine, octopamine, amphetamines) activate mouse, rat and northern treeshrew TAAR4 either to much lesser extent or not at all (data not shown).

### Convergent evolution of TAAR3 and TAAR4 in apes and *Callithrichinae*

One surprising discovery was that species carrying TAAR3 and TAAR4 pseudogenes significantly overlap (Figures 1, 2). These ORF disruptions very likely reflect a complete loss of function as there is no evidence for constraint in these disrupted genes. Some of those result from pseudogenization events on the same lineages in both receptors, as for e.g. in the common ancestor of humans, chimpanzees and gorillas and on the lineage to the white-handed gibbon. In the 2 marmosets the loss of constraint occurred in their common ancestor for TAAR3 and TAAR4 but inactivating mutations occurred and became fixed before or after the lineage split. Thus, TAAR3 and TAAR4 pseudogenization does not fully correlate, as also seen for the lineage to orangutan and the woolly monkey. However, both the partial overlap and the observed correlation of constraint among TAAR3 and TAAR4 in intact ORF lineages suggest that TAAR3 and TAAR4 have common constraint-determining factors, and probably similar functions. Our study suggests that agonists involved in this function should be active in all or at least many primates. Further research on the function of TAAR is required to uncover their comprehensive physiological functions in mammals.

### Conclusion

Our functional data for TAAR3, TAAR4 and TAAR5 revealed a high species-specificity for the identified volatile amine agonists. Since intact primate TAAR3–5 evolve under purifying selection we suggest that the identified mouse TAAR3–5 agonists are surrogate ligands but not the natural agonists. The correlated evolution of TAAR3 and TAAR4 suggest similar agonists and physiological functions for these 2 receptors.

More generally, the present study emphasizes the usefulness of investigating function as well as sequence evolution in a wide range of organisms to interpret functional studies in a single species.

## Supporting Information

Figure S1 Phylogenetic trees of primate species inferred from the combined TAAR3-TAAR4-TAAR5 sequence dataset. A:The evolutionary history of 21 primates was inferred using the Neighbor-Joining method [16]. The evolutionary distances were computed using the Maximum Composite Likelihood model [17] implemented in MEGA4 [15]. B: The phylogenetic relationship of 21 primates was inferred using the Maximum Likelihood method. The F84 model [18] was specified and analyses were conducted by using PHYLIP3.69 [19]. The bootstrap consensus trees inferred from 1,000 replicates are taken to represent the evolutionary history of the taxa analyzed [20]. The percentage of replicate trees in which the associated taxa clustered together in the bootstrap test (1,000 replicates) are shown next to the branches [20] The tree is drawn to scale, with branch length corresponding to nucleotide substitutions per site. All codon positions were included, all postions containing gaps and missing data were eliminated from the dataset. There were a total of 2289 nucleotides in the final dataset.
(0.96 MB TIF)

Figure S2 Phylogenetic trees of mammalian species inferred from the concatenated TAAR3-TAAR4-TAAR5 sequence dataset. A:The evolutionary history of 14 mammals was inferred using the Neighbor-Joining method [16]. The evolutionary distances were computed using the Maximum Composite Likelihood model [17] implemented in MEGA4 [15]. B: The phylogenetic relationship of 14 mammals was inferred using the Maximum Likelihood method. The F84 model [18] was specified and analyses were conducted by using PHYLIP3.69 [19]. The bootstrap consensus trees inferred from 1,000 replicates are taken to represent the evolutionary history of the 14 mammals analyzed [20]. The percentage of replicate trees in which the associated taxa clustered together in the bootstrap test (1,000 replicates) are shown next to the branches [20] The trees are drawn to scale, with branch length corresponding to nucleotide substitutions per site. All codon positions were included, all postions containing gaps and missing data were eliminated from the dataset. There were a total of 3063 nucleotides in the final dataset.
(0.65 MB TIF)

Figure S3 Primate TAAR3 pseudogenization. Events causing pseudogenes (indicated with ψ) are depicted in bold. TAAR3 is inactivated not only in apes except siamang but also in some New World monkeys.
(0.47 MB TIF)

Figure S4 Primate TAAR4 pseudogenization. TAAR4 is a pseudogene (ψ) in all apes except orangutan and siamang and in 3 New World monkeys. Positions hit by insertions, deletions or stop mutations are indicated in bold.
(0.58 MB TIF)

Figure S5 Primate TAAR5 pseudogenization. TAAR5 is a pseudogene in white- and yellow-cheeked gibbon and Philippine tarsier. All other primate TAAR5 possess an intact ORF. Nucleotide insertions or deletions causing pseudogenization (ψ) are depicted in bold.
(0.28 MB TIF)

Figure S6 Functional characterization of mouse TAAR4 using a CRE-SEAP reporter gene assay. HEK293 cells were transiently co-transfected with CRE-SEAP reporter plasmid (Clontech) and mouse TAAR4 and tested for agonist induced SEAP-activity. The basal value of non-stimulated mock-transfected HEK293 determined was 193,208±21,052 cpm/well. Data are given as mean±SEM of 2 independent experiments each performed in triplicates. Concentration-response curves of agonists were determined using GraphPad Prism.
(0.14 MB TIF)

Figure S7 Phylogenetic tree of 14 mammalian species. Phylogenetic tree is based on phylogeny described in [27]. d_N_/d_S_-ratios (ω) ratios for each branch using full length TAAR3 (A), TAAR4 (B) and TAAR5 (C) sequences of selected mammals were calculated by using a “free ratio” model implemented in PAML and are shown in italic above the respective branch. The number of non-synonymous and synonymous substitutions for each branch is shown in parentheses. Branch-site models were performed to detect positive selected sites in certain branches. Foreground branches are labeled with #.
(0.52 MB TIF)

Figure S8 Serpentine model of TAAR4 rhodopsin constructs. Amino acid sequence of mouse TAAR4 is shown. All constructs possess a N-terminal HA- and a C-terminal FLAG-tag (light gray). Each construct has additionally to its own N terminus the first 20 amino acids of bovine rhodopsin N terminus and a modified C terminus corresponding to 12 C-terminal amino acids of the rhesus monkey TAAR4 (depicted in dark gray). Amino acid positions differing between mouse and rat TAAR4 are shown in white.
(0.24 MB TIF)

Table S1 NCBI database accession numbers and sequence description.
(0.24 MB PDF)

Table S2 Sources of genomic DNA used for TAAR3, TAAR4 and TAAR5 amplification.
(0.13 MB PDF)

Table S3 Primers used for TAAR3, TAAR4 and TAAR5 ortholog amplification, sequencing and site-directed introduction of epitope tags.
(0.10 MB PDF)

Table S4 Phylogenetic trees in NEWICK notation.
(0.10 MB PDF)

Table S5 Structural comparison of mammalian GPCR orthologs. The amino acid sequence information of 8 full-length orthologs (*Bos taurus, Cavia porcellus, Echinops telfairi, Macaca mulatta, Mus musculus, Oryctolagus cuniculus, Rattus norvegicus, Sus scrofa*) of each receptor was used to determine the structural conservation between mammalian orthologs (given as % aa identity determined by ClustalW implemented in MegAlign of Lasergene 7.1.) are shown. Data are given as mean±S.D. ADRB1, beta-1-adrenergic receptor; ADRB2, beta-2-adrenergic receptor; MC4R, melancortin receptor 4; V2R, vasopressin type 2 receptor.
(0.05 MB PDF)

Table S6 Maximum likelihood estimates of d_N_/d_S_ ratios (ω) for primate and 14 non-primate mammalian TAAR3, TAAR4 and TAAR5 under different models using PAML.
(0.19 MB PDF)

Table S7 Likelihood ratio test (LRT) statistics for testing site-specific models within primate ORF lineages and 14 non-primate mammalians.
(0.15 MB PDF)
