# Supplementary figures and images for: Structural and Functional Evolution of the Trace Amine-Associated Receptors TAAR3, TAAR4 and TAAR5 in Primates
Source: PLoS One. 2010 Jun 15;5(6):e11133. doi: 10.1371/journal.pone.0011133 (PMC2886124; doi:10.1371/journal.pone.0011133)

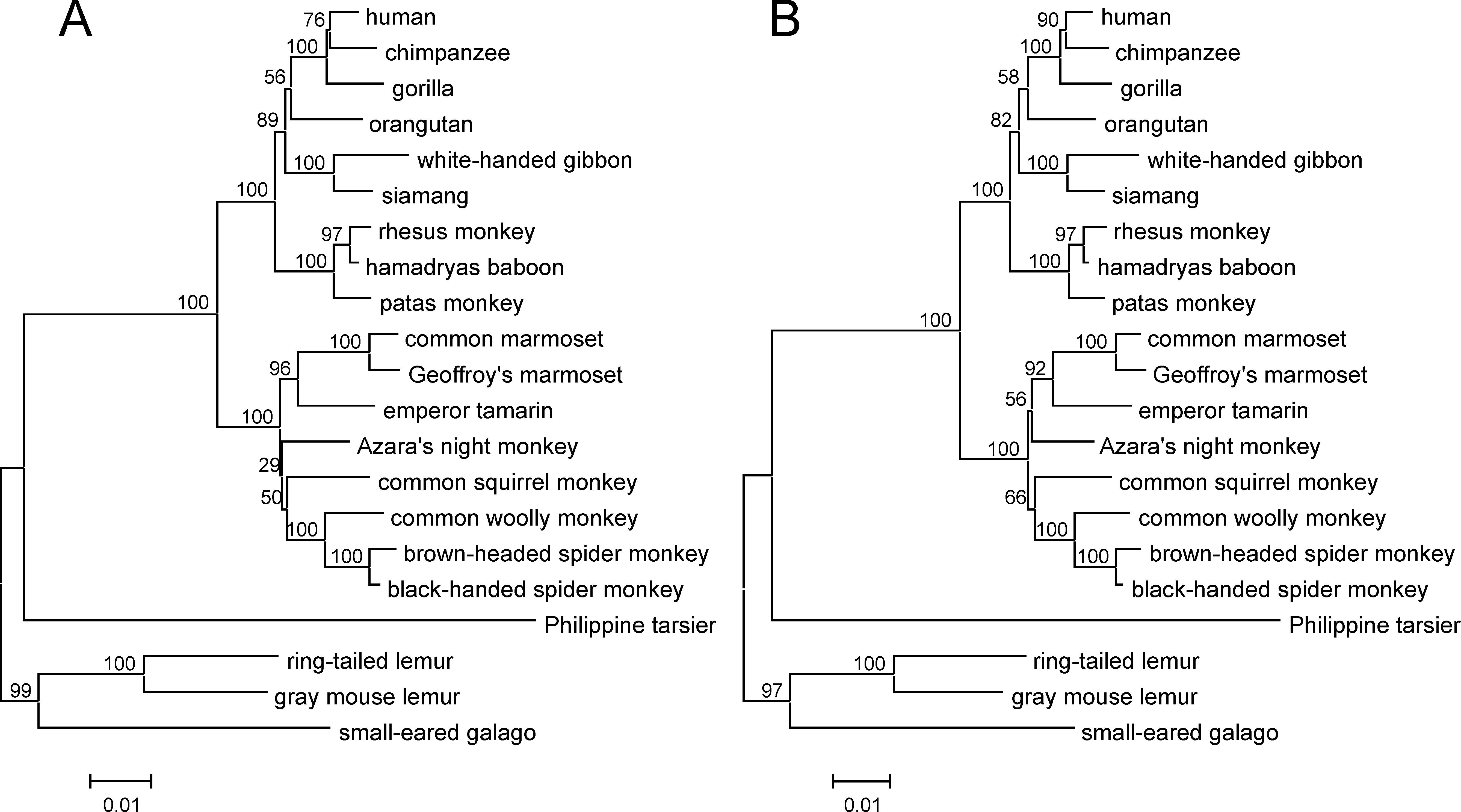

Supplement: Figure S1 — Phylogenetic trees of primate species inferred from the combined TAAR3-TAAR4-TAAR5 sequence dataset. A:The evolutionary history of 21 primates was inferred using the Neighbor-Joining method [16]. The evolutionary distances were computed using the Maximum Composite Likelihood model [17] implemented in MEGA4 [15]. B: The phylogenetic relationship of 21 primates was inferred using the Maximum Likelihood method. The F84 model [18] was specified and analyses were conducted by using PHYLIP3.69 [19]. The bootstrap consensus trees inferred from 1,000 replicates are taken to represent the evolutionary history of the taxa analyzed [20]. The percentage of replicate trees in which the associated taxa clustered together in the bootstrap test (1,000 replicates) are shown next to the branches [20] The tree is drawn to scale, with branch length corresponding to nucleotide substitutions per site. All codon positions were included, all postions containing gaps and missing data were eliminated from the dataset. There were a total of 2289 nucleotides in the final dataset. (0.96 MB TIF) [file pone.0011133.s001.tif]

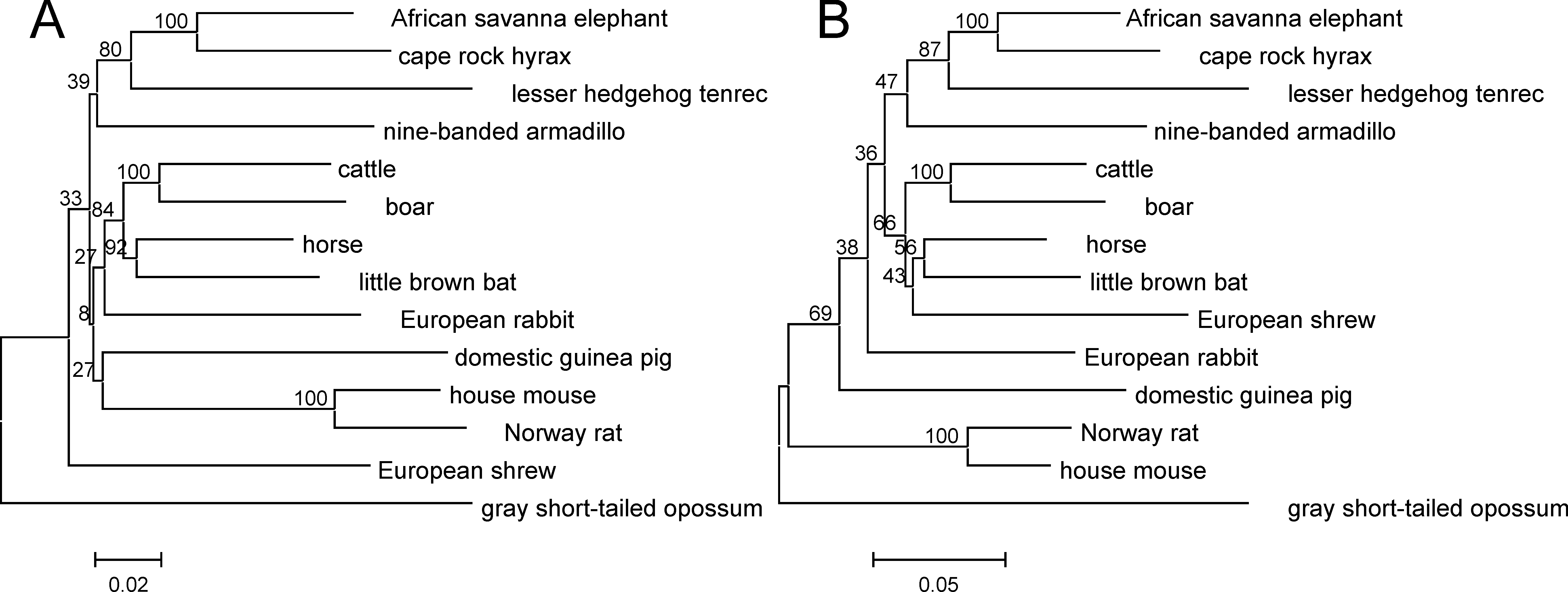

Supplement: Figure S2 — Phylogenetic trees of mammalian species inferred from the concatenated TAAR3-TAAR4-TAAR5 sequence dataset. A:The evolutionary history of 14 mammals was inferred using the Neighbor-Joining method [16]. The evolutionary distances were computed using the Maximum Composite Likelihood model [17] implemented in MEGA4 [15]. B: The phylogenetic relationship of 14 mammals was inferred using the Maximum Likelihood method. The F84 model [18] was specified and analyses were conducted by using PHYLIP3.69 [19]. The bootstrap consensus trees inferred from 1,000 replicates are taken to represent the evolutionary history of the 14 mammals analyzed [20]. The percentage of replicate trees in which the associated taxa clustered together in the bootstrap test (1,000 replicates) are shown next to the branches [20] The trees are drawn to scale, with branch length corresponding to nucleotide substitutions per site. All codon positions were included, all postions containing gaps and missing data were eliminated from the dataset. There were a total of 3063 nucleotides in the final dataset. (0.65 MB TIF) [file pone.0011133.s002.tif]

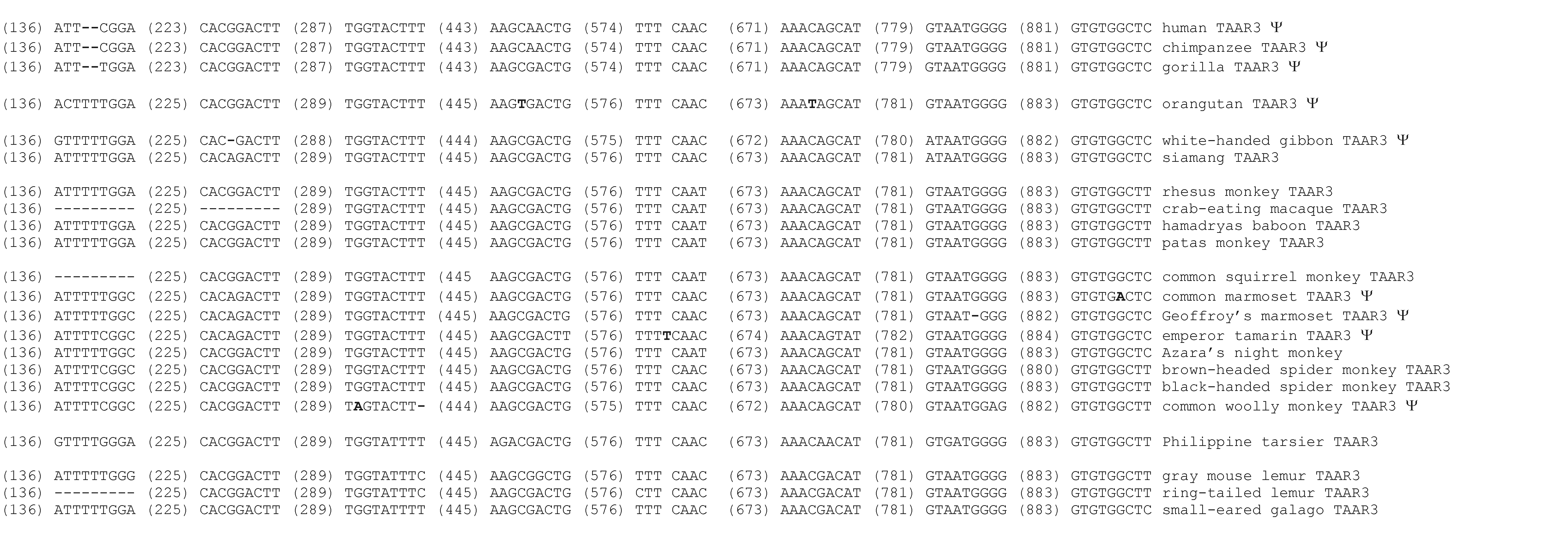

Supplement: Figure S3 — Primate TAAR3 pseudogenization. Events causing pseudogenes (indicated with ψ) are depicted in bold. TAAR3 is inactivated not only in apes except siamang but also in some New World monkeys. (0.47 MB TIF) [file pone.0011133.s003.tif]

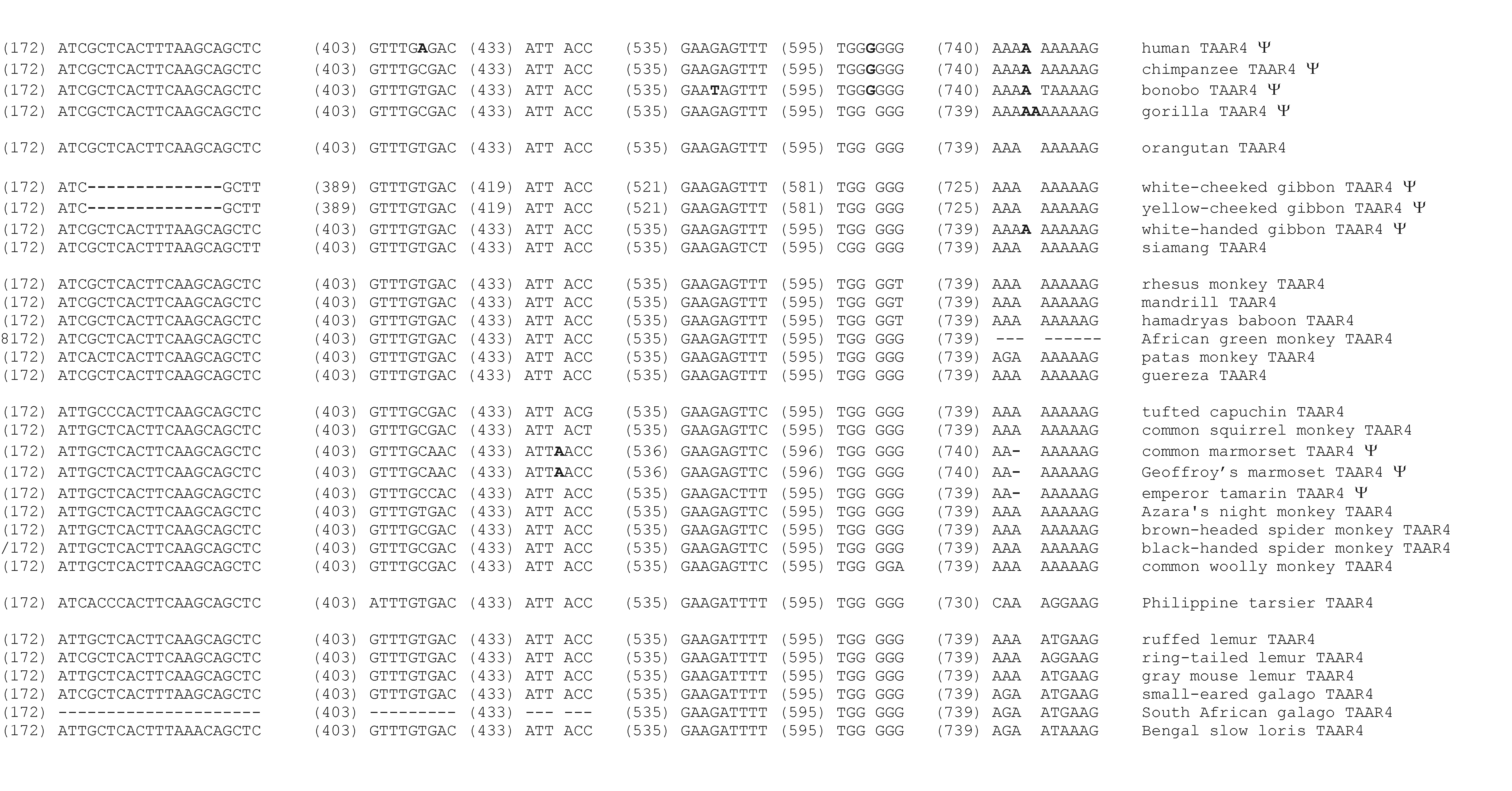

Supplement: Figure S4 — Primate TAAR4 pseudogenization. TAAR4 is a pseudogene (ψ) in all apes except orangutan and siamang and in 3 New World monkeys. Positions hit by insertions, deletions or stop mutations are indicated in bold. (0.58 MB TIF) [file pone.0011133.s004.tif]

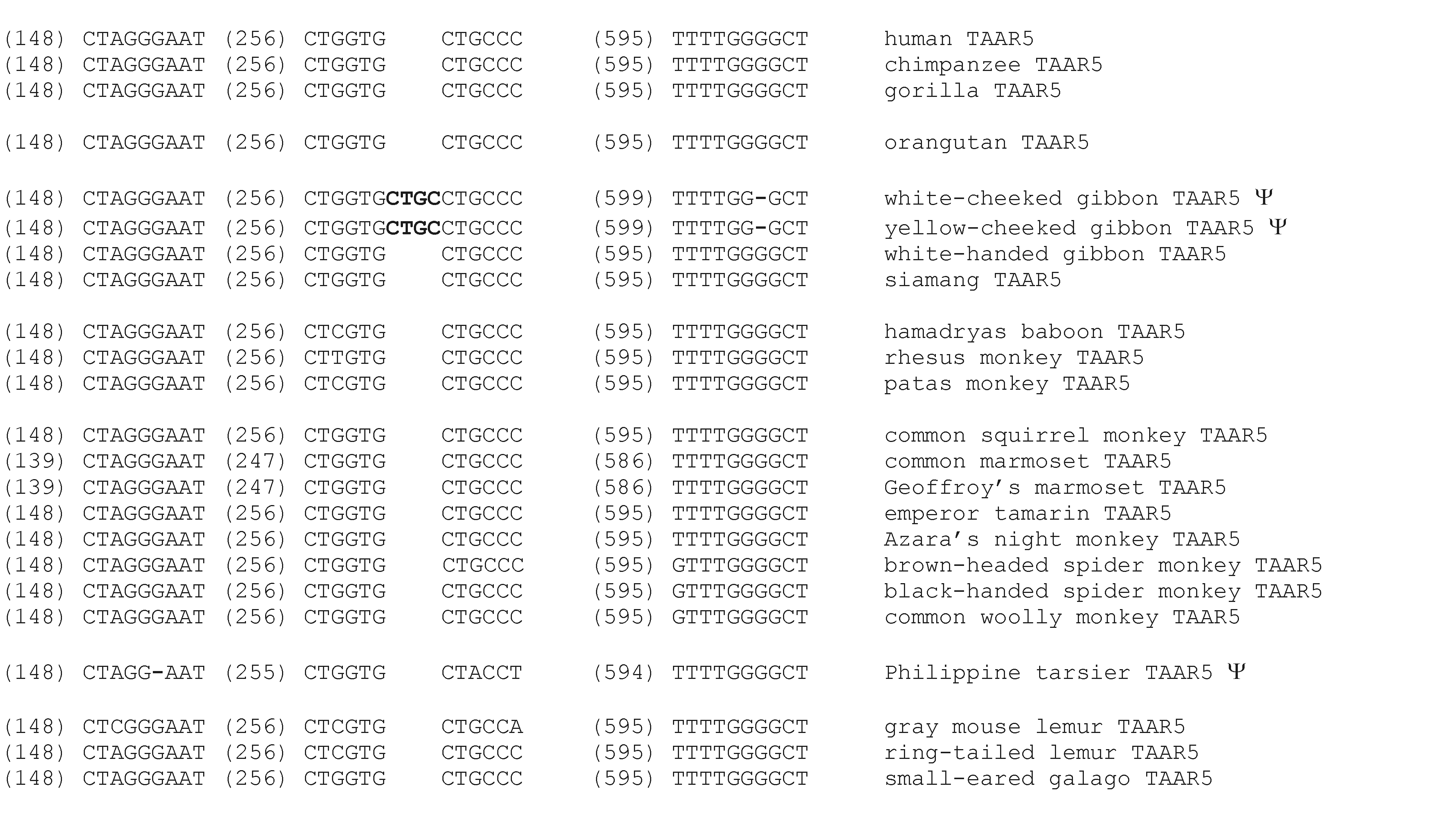

Supplement: Figure S5 — Primate TAAR5 pseudogenization. TAAR5 is a pseudogene in white- and yellow-cheeked gibbon and Philippine tarsier. All other primate TAAR5 possess an intact ORF. Nucleotide insertions or deletions causing pseudogenization (ψ) are depicted in bold. (0.28 MB TIF) [file pone.0011133.s005.tif]

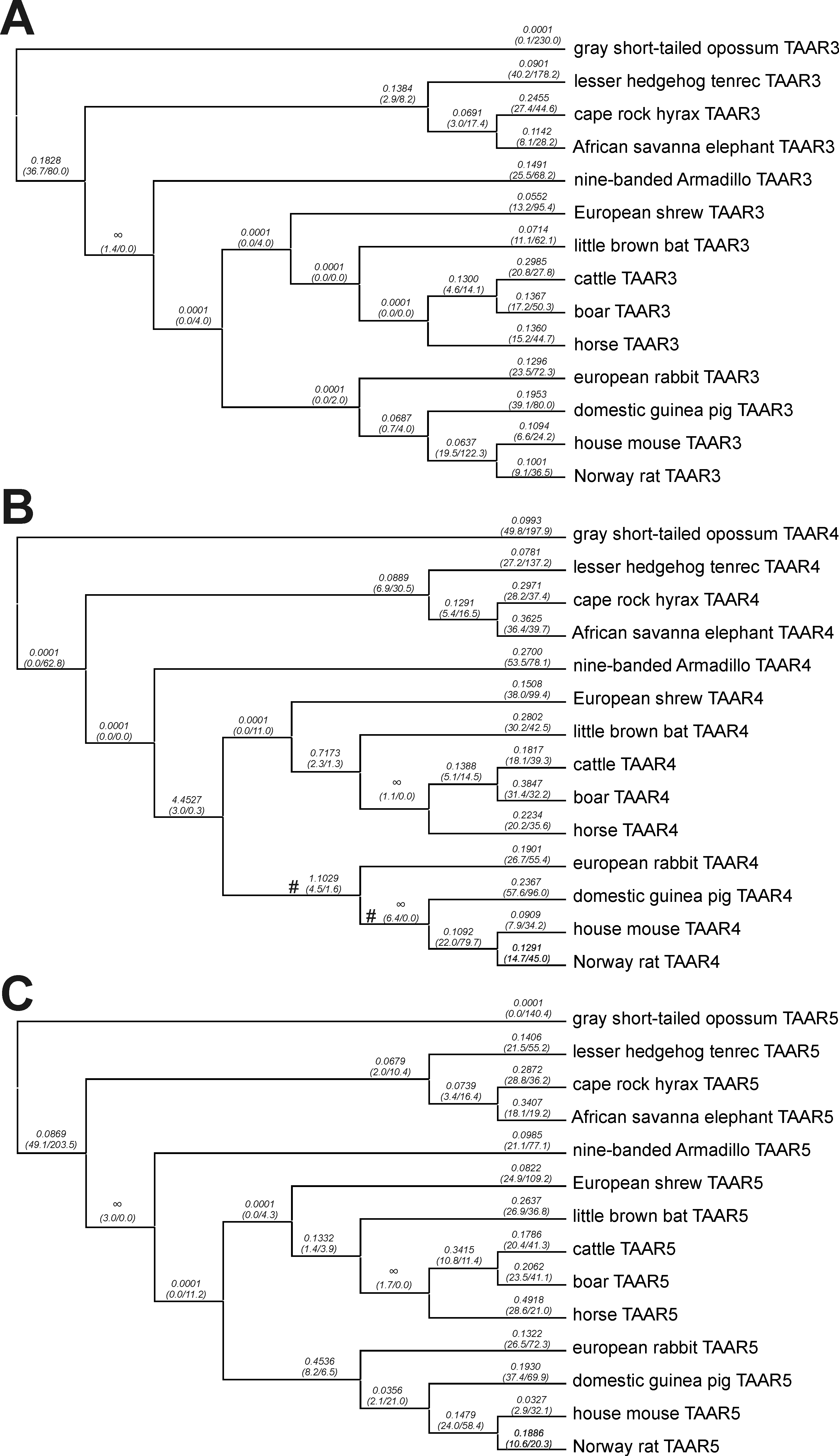

Supplement: Figure S7 — Phylogenetic tree of 14 mammalian species. Phylogenetic tree is based on phylogeny described in [27]. dN/dS-ratios (ω) ratios for each branch using full length TAAR3 (A), TAAR4 (B) and TAAR5 (C) sequences of selected mammals were calculated by using a “free ratio” model implemented in PAML and are shown in italic above the respective branch. The number of non-synonymous and synonymous substitutions for each branch is shown in parentheses. Branch-site models were performed to detect positive selected sites in certain branches. Foreground branches are labeled with #. (0.52 MB TIF) [file pone.0011133.s007.tif]

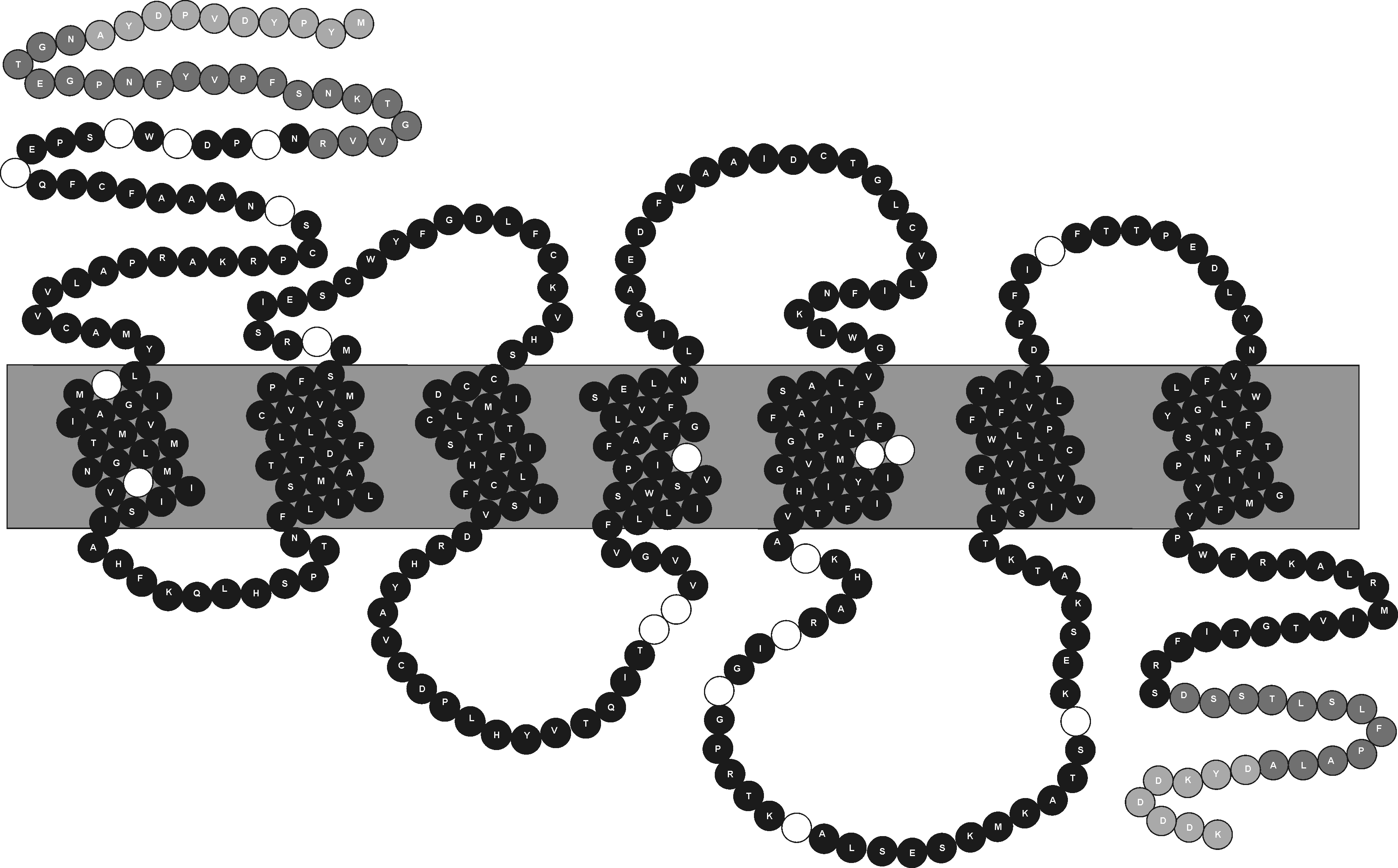

Supplement: Figure S8 — Serpentine model of TAAR4 rhodopsin constructs. Amino acid sequence of mouse TAAR4 is shown. All constructs possess a N-terminal HA- and a C-terminal FLAG-tag (light gray). Each construct has additionally to its own N terminus the first 20 amino acids of bovine rhodopsin N terminus and a modified C terminus corresponding to 12 C-terminal amino acids of the rhesus monkey TAAR4 (depicted in dark gray). Amino acid positions differing between mouse and rat TAAR4 are shown in white. (0.24 MB TIF) [file pone.0011133.s008.tif]
