# Supplementary material for: Structural and Functional Evolution of the Trace Amine-Associated Receptors TAAR3, TAAR4 and TAAR5 in Primates
Source: PLoS One. 2010 Jun 15;5(6):e11133. doi: 10.1371/journal.pone.0011133 (PMC2886124; doi:10.1371/journal.pone.0011133)
Supplement: Table S1 — NCBI database accession numbers and sequence description. (0.24 MB PDF) [file pone.0011133.s009.pdf]

| | | TAAR3 length | TAAR3 source | TAAR4 length | TAAR4 source | TAAR5 length | TAAR5 source |
|---|---|---|---|---|---|---|---|
| ***Mammalia*** | | | | | | | |
| ***Protheria*** | | | | | | | |
| *Ornithorhynchus anatinus* | platypus | full length | NCBI *Ornithorhynchus anatinus* trace archive: gnl\|ti\|750149729 and others | full length | NCBI *Ornithorhynchus anatinus* trace archive: gnl\|ti\|1246961726 and others | | |
| ***Metatheria*** | | | | | | | |
| *Monodelphis domestica** | gray short-tailed opossum | full length | NCBI *Monodelphis domestica* trace archive: gnl\|ti\|397122803 and others | full length 3x | NCBI *Monodelphis domestica* trace archive: gnl\|ti\|473024684 and others | full length | NCBI *Monodelphis domestica* trace archive: gnl\|ti\|343429597 and others |
| *Macropus eugenii* | tammar wallaby | full length | NCBI *Macropus eugenii* trace archive: gnl\|ti\|1378047881 and others | full length | NCBI *Macropus eugenii* trace archive: gnl\|ti\|1275406430 and others | aa8-end | NCBI *Macropus eugenii* trace archive: gnl\|ti\|976302676 |
| ***Eutheria*** | | | | | | | |
| ***Afrotheria*** | | | | | | | |
| *Procavia capensis** | cape rock hyrax | full length | NCBI *Procavia capensis* trace archive: gnl\|ti\|1203593751 and others | full length 3x | NCBI accession: FJ372519, NCBI *Procavia capensis* trace archive: gnl\|ti\|1203477241 and others | full length | NCBI *Procavia capensis* trace archive: gnl\|ti\|1295000815 and others |
| *Loxodonta africana** | African savanna elephant | full length | NCBI *Loxodonta africana* trace archive: gnl\|ti\|1714433321 and others | full length 2x | NCBI accession: FJ372488 and NCBI *Loxodonta africana* trace archive: gnl\|ti\|1695377756 and others | full length | NCBI *Loxodonta africana* trace archive: gnl\|ti\|1865757347 and others |
| *Elephas maximus* | asiatic elephant | | | aa63-317 | NCBI accession: FJ372465 | | |
| *Trichechus manatus* | Caribbean manatee | | | aa13-315 (P) | NCBI accession: FJ372528 | | |
| *Echinops telfairi** | lesser hedgehog tenrec | full length | NCBI *Echinops telfairi* trace archive: gnl\|ti\|682625873 and others | full length 2x (1P) | NCBI *Echinops telfairi* trace archive: gnl\|ti\|688316620 and others | full length | NCBI *Echinops telfairi* trace archive: gnl\|ti\|659227110 and others |
| *Orycteropus afer* | aardvark | | | aa14-310 | NCBI accession: FJ372504, FJ375205 | | |

| | | | | | | | |
|---|---|---|---|---|---|---|---|
| ***Xenarthra*** *Bradypus tridactylus* | pale-throated sloth | | | aa14-299 | NCBI accession: FJ372454 | | |
| *Dasypus novemcinctus** | nine-banded Armadillo | full length | NCBI *Dasypus novemcinctus* trace archive: gnl\|ti\|601523115 and others | full length | NCBI *Dasypus novemcinctus* trace archive: gnl\|ti\|567276505 and others | full length | NCBI *Dasypus novemcinctus* trace archive: gnl\|ti\|1810404270 and others |
| *Choloepus hoffmanni* | Hoffmann's two-fingered sloth | aa1-87 and aa219-end | NCBI *Choloepus hoffmanni* trace archive: gnl\|ti\|1316582719 and others | full length | NCBI *Choloepus hoffmanni* trace archive: gnl\|ti\|1357779584 and others | full length | NCBI *Choloepus hoffmanni* trace archive: gnl\|ti\|1315053175 and others |
| ***Laurasiatheria Insectivora*** *Erinaceus concolor* | eastern European hedgehog | | | aa62-312 | NCBI accession: FJ372468 | | |
| *Erinaceus europaeus* | western European hedgehog | aa53-end | NCBI *Erinaceus europaeus* trace archive: gnl\|ti\|910108268 and others | full length 2x (1P) | NCBI *Erinaceus europaeus* trace archive: gnl\|ti\|932971697 and others | full length | NCBI *Erinaceus europaeus* trace archive: gnl\|ti\|970975651 and others |
| *Sorex araneus** | European shrew | full length | NCBI *Sorex araneus* trace archive: gnl\|ti\|885213269 and others | full length 2x (1P) | NCBI *Sorex araneus* trace archive: gnl\|ti\|893813953 and others | full length | NCBI *Sorex araneus* trace archive: gnl\|ti\|892168220 and others |
| *Talpa europaea* | European mole | | | aa60-259 | NCBI accession: FJ372525 | | |
| ***Chiroptera*** *Pteropus sp.* | flying fox | | | aa14-303 | NCBI accession: FJ372520 | | |
| *Pteropus vampyrus* | large flying fox | aa1-140 and aa284-end | NCBI *Pteropus vampyrus* trace archive: gnl\|ti\|1399038458 and others | full length | NCBI *Pteropus vampyrus* trace archive: gnl\|ti\|1329134482 and others | full length | NCBI *Pteropus vampyrus* trace archive: gnl\|ti\|1384511508 and others |
| *Myotis albescens* | silver-tipped myotis | | | aa9-322 | NCBI accession: FJ372498 | | |
| *Myotis lucifugus** | little brown bat | full length | NCBI *Myotis lucifugus* trace archive: gnl\|ti\|1912741925 and others | full length | NCBI *Myotis lucifugus* trace archive: gnl\|ti\|1899487221and others | full length | NCBI *Myotis lucifugus* trace archive: gnl\|ti\|1895512953 and others |

| | | | | | | | |
|---|---|---|---|---|---|---|---|
| *Rhogeessa io* | Thomas's yellow bat | | | aa14-312 | NCBI accession: FJ372522 | | |
| ***Cetartiodactyla*** | | | | | | | |
| *Hexaprotodon liberiensis* | pygmy hippopotamus | | | aa13-315 | NCBI accession: FJ372475 | | |
| *Bos taurus** | cattle | full length | NCBI accession: FJ372446 and NCBI *Bos taurus* trace archive: gnl\|ti\|501216465 and others | full length | NCBI *Bos taurus* trace archive: gnl\|ti\|620739238 and others | full length | NCBI accession: FJ372540 and NCBI *Bos taurus* trace archive: gnl\|ti\|508561082 and others |
| *Bison bonasus* | European bison | | | aa100-290 | NCBI accession: FJ372453 | | |
| *Ovis aries* | sheep | | | aa14-304 | NCBI accession: FJ372507 | | |
| *Ovibos moschatus* | muskox | | | aa71-312 | NCBI accession: FJ372506 | | |
| *Hippotragus niger* | sable antelope | | | aa42-314 | NCBI accession: FJ372476 | | |
| *Cervus elaphus* | red deer | | | aa14-304 | NCBI accession: FJ372462 | | |
| *Giraffa camelopardalis* | giraffe | | | aa67-312 | NCBI accession: FJ372472 | | |
| *Sus scrofa** | boar | full length | NCBI *Sus scrofa* trace archive: gnl\|ti\|1420532685 and others | full length | NCBI *Sus scrofa* trace archive: gnl\|ti\|1579919251 and others | full length | NCBI *Sus scrofa* trace archive: gnl\|ti\|1579918704 and others |
| *Camelus bactrianus* | bactrian camel | | | aa75-312 | NCBI accession: FJ372455 | | |
| *Vicugna vicugna* | Vicugna | full length | NCBI *Vicugna vicugna* trace archive: gnl\|ti\|1983213061 and others | full length | NCBI *Vicugna vicugna* trace archive: gnl\|ti\|1963834526 and others | aa28-end | NCBI *Vicugna vicugna* trace archive: gnl\|ti\|1930070997 and others |
| ***Perissodactyla*** | | | | | | | |
| *Equus caballus** | horse | full length | NCBI *Equus caballus* trace archive: gnl\|ti\|1232113054 and others | full length | NCBI *Equus caballus* trace archive: gnl\|ti\|1234728610 and others | full length 2x | NCBI *Equus caballus* trace archive: gnl\|ti\|1204042749 and others |
| *Equus przewalskii* | Przewalski horse | | | aa10-335 | NCBI accession: FJ372466, FJ372467 | | |
| *Ceratotherium simum* | white rhinoceros | | | aa13-306 | NCBI accession: FJ372460 | | |

| | | | | | | | |
|---|---|---|---|---|---|---|---|
| *Tapirus sp.* | tapir | | | aa13-314 | NCBI accession: FJ372526, FJ372527 | | |
| ***Carnivora*** | | | | | | | |
| *Feliformia* | | | | | | | |
| *Hyaena hyaena* | striped hyena | | | aa21-304 | NCBI accession: FJ372479 | | |
| *Felis catus* | cat | aa106-end | NCBI *Felis catus* trace archive: gnl\|ti\|652118115 and others | full length | NCBI accession: FJ372470 and NCBI *Felis catus* trace archive: gnl\|ti\|965531899 and others | full length | NCBI *Felis catus* trace archive: gnl\|ti\|653605578 and others |
| *Acinonyx jubatus* | cheetah | | | aa14-312 | NCBI accession: FJ372449 | | |
| *Panthera leo* | lion | | | aa15-316 | NCBI accession: FJ372508 | | |
| *Panthera onca* | jaguar | | | aa15-314 | NCBI accession: FJ372509 | | |
| *Panthera tigris* | tiger | | | aa14-312 | NCBI accession: FJ372510 | | |
| *Uncia uncia* | snow leopard | | | aa21-312 | NCBI accession: FJ372530, FJ372531 | | |
| *Carniformia* | | | | | | | |
| *Zalophus californianus* | California sea lion | | | aa13-317 (P) | NCBI accession: FJ372538 | | |
| *Phoca sibirica* | Baikal seal | | | aa12-307 | NCBI accession: FJ372516 | | |
| *Nasua nasua* | ring-tailed coati | | | aa12-318 | NCBI accession: FJ372499 | | |
| *Mustelidae* | | | | | | | |
| *Lutra lutra* | Eurasian river otter | | | aa13-314 | NCBI accession: FJ372489, FJ372490 | | |
| *Martes foina* | beach marten | | | aa13-314 | NCBI accession: FJ372494 | | |
| *Ursidae* | | | | | | | |
| *Ailuropoda melanoleuca* | giant panda | | | aa12-315 | NCBI accession: FJ372450 | | |
| *Ursus malayanus* | Malayan sun bear | | | aa12-312 | NCBI accession: FJ372534 | | |
| *Ursus americanus* | American black bear | | | aa12-310 | NCBI accession: FJ372532 | | |
| *Ursus arctos* | brown bear | | | aa12-299 | NCBI accession: FJ372533 | | |
| *Ursus maritimus* | polar bear | | | aa12-310 | NCBI accession: FJ372535 | | |

| | | | | | | | |
|---|---|---|---|---|---|---|---|
| *Canidae*<br>*Canis lupus* | gray wolf | | | aa13-300 | NCBI accession: FJ372457 | | |
| *Canis familiaris* | dog | | | full length | NCBI accession: FJ372456 and NCBI *Canis familiaris* trace archive: gnl\|ti\|302723376 and others | full length | NCBI accession: FJ372543 and NCBI *Canis familiaris* trace archive: gnl\|ti\|290457734 and others |
| *Nyctereutes procyonoides* | raccoon dog | | | aa14-312 | NCBI accession: FJ372500 | | |
| *Vulpes vulpes* | red fox | | | aa14-312 | NCBI accession: FJ372537 | | |
| ***Euarchontoglires***<br>***Scandentia***<br>*Tupaia belangeri* | northern tree shrew | aa1-216 | NCBI *Tupaia belangeri* trace archive: gnl\|ti\|1069820554 and others | full length 2x | NCBI accession: FJ372529 and NCBI *Tupaia belangeri* trace archive: gnl\|ti\|1046628937 and others | aa204-end | NCBI *Tupaia belangeri* trace archive: gnl\|ti\|1072248002 and others |
| ***Glires***<br>*Lagomorpha*<br>*Ochotonidae*<br>*Ochotona princeps* | american Pika | full length (P) | NCBI *Ochotona princeps* trace archive: gnl\|ti\|1535960125 and others | aa177-end | NCBI *Ochotona princeps* trace archive: gnl\|ti\|1539847207 and others | full length | NCBI *Ochotona princeps* trace archive: gnl\|ti\|1528160173 and others |
| *Leporidae*<br>*Oryctolagus cuniculus** | european rabbit | full length | NCBI *Oryctolagus cuniculus* trace archive: gnl\|ti\|1992167154 and others | full length | NCBI *Oryctolagus cuniculus* trace archive: gnl\|ti\|638702844 and others | full length | NCBI *Oryctolagus cuniculus* trace archive: gnl\|ti\|621098157 and others |
| *Rodentia*<br>*Hystricognathi*<br>*Cavia porcellus** | domestic guinea pig | full length | NCBI *Cavia porcellus* trace archive: gnl\|ti\|1653413717 and others | full length 2x | NCBI *Cavia porcellus* trace archive: gnl\|ti\|1596694171 and others | full length 3x (2P) | NCBI *Cavia porcellus* trace archive: gnl\|ti\|1633904227 and others |
| *Octodontomys gliroides* | Mountain Degu | | | aa33-307 | NCBI accession: FJ372502 | | |

| | | | | | | | |
|---|---|---|---|---|---|---|---|
| *Sciurognathi* *Dipodomys ordii* | Ord's kangaroo rat | aa41-303 | NCBI *Dipodomys ordii* trace archive: gnl\|ti\|1569585119 and others | full length | NCBI *Dipodomys ordii* trace archive: gnl\|ti\|1586901258 and others | full length | NCBI *Dipodomys ordii* trace archive: gnl\|ti\|1558832023 and others |
| *Ondatra zibethicus* | muskrat | | | aa14-296 | NCBI accession: FJ372503 | | |
| *Meriones meridianus* | mid-day jird | | | aa12-311 | NCBI accession: FJ372495 | | |
| *Meriones unguiculatus* | Mongolian gerbil | | | aa12-311 | NCBI accession: FJ372496 | | |
| *Mus musculus** | house mouse | full length | NCBI accession: FJ372434, NM_001008429 | full length | NCBI accession: FJ372497, NM_001008499 | full length | NCBI accession: FJ372555, NM_001009574 |
| *Rattus norvegicus** | Norway rat | full length | NCBI accession: FJ372433, NM_001009532 | full length | NCBI accession: FJ372521, NM_175583 | full length | NCBI accession: FJ372562, NM_001009650 |
| *Spermophilus tridecemlineatus* | thirteen-lined ground squirrel | full length | NCBI *Spermophilus tridecemlineatus* trace archive: gnl\|ti\|1001097323 and others | aa221-end | NCBI *Spermophilus tridecemlineatus* trace archive: gnl\|ti\|1020590663 | aa118-end | NCBI *Spermophilus tridecemlineatus* trace archive: gnl\|ti\|1001118269 and others |
| ***Primates*** *Strepsirrhini* *Chiromyiformes* *Nycticebus bengalensis* | Bengal slow loris | | | aa44-312 | NCBI accession: FJ372501 | | |
| *Lemuriformes* *Microcebus murinus* | gray mouse lemur | full length | NCBI *Microcebus murinus* trace archive: gnl\|ti\|1556207801 and others | full length | NCBI *Microcebus murinus* trace archive: gnl\|ti\|1563038889 and others | full length | NCBI *Microcebus murinus* trace archive: gnl\|ti\|1570525518 and others |
| *Lemur catta* | ring-tailed lemur | aa75-330 | NCBI accession: FJ931108 | aa14-315 | NCBI accession: FJ372486, FJ372487 | aa8-333 | NCBI accession: FJ372554 |
| *Varecia variegata* | ruffed lemur | | | aa12-299 | NCBI accession: FJ372536 | | |

| | | | | | | | |
|---|---|---|---|---|---|---|---|
| *Lorisiformes*<br>*Otolemur garnettii* | small-eared galago | full length | NCBI *Otolemur garnettii* trace archive: gnl\|ti\|1095202340 and others | aa42-end | NCBI *Otolemur garnettii* trace archive: gnl\|ti\|2016312907 and others | aa19-end | NCBI *Otolemur garnettii* trace archive: gnl\|ti\|1094613047 and others |
| *Galago moholi* | South African galago | | | aa158-311 | NCBI accession: FJ372471 | | |
| *Haplorrhini*<br>*Tarsiiformes*<br>*Tarsius syrichta* | Philippine tarsier | full length | NCBI *Tarsius syrichta* trace archive: gnl\|ti\|1551473575 and others | full length | NCBI *Tarsius syrichta* trace archive: gnl\|ti\|1546582642 and others | full length (P) | NCBI *Tarsius syrichta* trace archive: gnl\|ti\|1496118726 and others |
| *Simiiformes*<br>*Platyrrhini*<br>*Aotidae*<br>*Aotus azarai* | Azara's Night Monkey | aa34-326 | NCBI accession: FJ931100 | aa26-312 | NCBI accession: FJ372451 | aa16-321 | NCBI accession: FJ931101 |
| *Atelidae*<br>*Lagothrix lagotricha* | common woolly monkey | aa21-336 (P) | NCBI accession: FJ931109 | aa14-316 | NCBI accession: FJ372485 | aa28-320 | NCBI accession: FJ931110 |
| *Ateles fusciceps* | brown-headed spider monkey | aa28-327 | NCBI accession: FJ931102 | aa1-314 | NCBI accession: FJ372452 | aa28-320 | NCBI accession: FJ931103 |
| *Ateles geoffroyi* | black-handed spider monkey | aa18-340 | NCBI accession: FJ931104 | aa40-314 | NCBI accession: FJ931105 | aa19-end | NCBI accession: FJ931106 |
| *Cebidae*<br>*Callithrix jacchus* | common marmoset | full length (P) | NCBI *Callithrix jacchus* trace archive: gnl\|ti\|1129809010 and others | full length (P) | NCBI *Callithrix jacchus* trace archive: gnl\|ti\|1133228298 and others | full length | NCBI *Callithrix jacchus* trace archive: gnl\|ti\|1143837426 and others |
| *Callithrix geoffroyi* | Geoffroy's marmoset | aa16-340 (P) | NCBI accession: FJ372447, FJ372448 | aa11-320 (P) | NCBI accession: FJ931107 | aa6-end | NCBI accession: FJ372541, FJ372542 |
| *Saguinus imperator* | emperor tamarin | aa18-342 (P) | NCBI accession: FJ372426, FJ372427 | aa12-313 (1P) | NCBI accession: FJ931111, FJ931112 | aa29-318 | NCBI accession: FJ931113 |
| *Cebus apella* | tufted capuchin | | | aa26-296 | NCBI accession: FJ372458, FJ372459 | | |

| | | | | | | | |
|---|---|---|---|---|---|---|---|
| *Saimiri sciureus* | common squirrel monkey | aa74-327 | NCBI accession: FJ931114 | full length | NCBI accession: FJ372523, FJ372524 and EF549700 | aa16-329 | NCBI accession: FJ931115 |
| *Catarrhini*<br>*Cercopithecoidea*<br>*Erythrocebus patas* | patas monkey | aa15-340 | NCBI accession: FJ372444, FJ372445 | aa11-315 | NCBI accession: FJ372469 | aa20-end | NCBI accession: FJ372544 |
| *Macaca mulatta* | rhesus monkey | full length | NCBI accession: FJ372435, FJ372436 and NCBI *Macaca mulatta* trace archive: gnl\|ti\|376527395 and others | full length | NCBI accession: FJ372491, FJ372492 and NCBI *Macaca mulatta* trace archive: gnl\|ti\|486761772 and others | full length | NCBI *Macaca mulatta* trace archive: gnl\|ti\|349474498 and others |
| *Macaca fascicularis* | crab-eating Macaque | aa88-340 | NCBI accession: FJ372437 | | | | |
| *Mandrillus sphinx* | mandrill | | | aa12-312 | NCBI accession: FJ372493 | | |
| *Papio hamadryas* | hamadryas baboon | full length | NCBI accession: FJ372430, FJ372431 and NCBI *Papio hamadryas* trace archive: gnl\|ti\|2010511994 and others | full length | NCBI accession: FJ372515, FJ372539 and NCBI *Papio hamadryas* trace archive: gnl\|ti\|1959076673 and others | full length | NCBI accession: FJ372558, FJ372559 and NCBI *Papio hamadryas* trace archive: gnl\|ti\|1998875785 and others |
| *Cercopithecus aethiops* | African green monkey | | | aa26-221 | NCBI accession: FJ372461 | | |
| *Colobus guereza* | guereza | | | aa10-314 | NCBI accession: FJ372463, FJ372464 | | |
| *Hominoidea*<br>*Hylobatidae*<br>*Hylobates lar* | white-handed gibbon | aa16-337 (P) | NCBI accession: FJ372440, FJ372441 | aa18-314 | NCBI accession: FJ372481 | aa14-end (P) | NCBI accession: FJ372549, FJ372550 |
| *Nomascus gabriellae* | red-cheeked Gibbon | | | aa11-322 (P) | NCBI accession: FJ372480 | aa16-323 (P) | NCBI accession: FJ372548 |

| | | | | | | | |
|---|---|---|---|---|---|---|---|
| *Nomascus leucogenys* | white-cheeked Gibbon | | | full length (P) | NCBI accession: FJ372482, FJ372483 and NCBI *Nomascus leucogenys* trace archive: gnl\|ti\|2088070237 and others | full length (P) | NCBI accession: FJ372551 and NCBI *Nomascus leucogenys* trace archive: gnl\|ti\|2092296901 and others |
| *Symphalangus syndactylus* | siamang | aa16-340 (P) | NCBI accession: FJ372438, FJ372439 | aa12-317 | NCBI accession: FJ372484 | aa9-334 | NCBI accession: FJ372552, FJ372553 |
| *Hominidae* | | | | | | | |
| *Gorilla gorilla* | Western Gorilla | aa15-end (P) | NCBI accession: FJ372442, FJ372443 and NCBI *Gorilla gorilla* trace archive: gnl\|ti\|1669650693 and others | full length (P) | NCBI accession: FJ372473, FJ372474 and NCBI *Gorilla gorilla* trace archive: gnl\|ti\|2033155805 and others | full length | NCBI accession: FJ372545, FJ372546 and NCBI *Gorilla gorilla* trace archive: gnl\|ti\|1688237874 and others |
| *Homo sapiens* | human | full length (P) | NCBI accession: NG_002481 | full length (P) | NCBI accession: FJ372477, FJ372478, NG_004855 | full length | NCBI accession: FJ372547, NM_003967 |
| *Pan paniscus* | bonobo | | | aa29-313 (P) | NCBI accession: FJ372511, FJ372512 | | |
| *Pan troglodytes* | chimpanzee | full length (P) | NCBI accession: FJ372432, NG_004781 | full length (P) | NCBI accession: FJ372513, FJ372514, AY702310 | full length | NCBI accession: FJ372556, FJ372557, NM_001009126 |
| *Pongo pygmaeus* | orangutan | full length (P) | NCBI accession: FJ372428, FJ372429 and NCBI *Pongo pygmaeus abelii* trace archive: gnl\|ti\|940938990 and others | full length (P) | NCBI accession: FJ372517, FJ372518 and NCBI *Pongo pygmaeus abelii* trace archive: gnl\|ti\|886956191 and others | full length | NCBI accession: FJ372560, FJ372561 and NCBI *Pongo pygmaeus abelii* trace archive: gnl\|ti\|1000874246 and others |
| ***Sauropsida*** | | | | | | | |
| *Anolis carolinensis* | green anole | | | | | full length | NCBI *Anolis carolinensis* trace archive: gnl\|ti\|1368146017 and others |

| | | | | | | | |
|---|---|---|---|---|---|---|---|
| *Gallus gallus* | chicken | | | | | full length | NCBI *Gallus gallus* trace archive: gnl\|ti\|282469281 and others |

Abbreviations: aa amino acid, (P) pseudogene, 14 sequences used for mammalian tree (Figure S2 and S7) are depicted with *
