# Supplementary material for: Structural and Functional Evolution of the Trace Amine-Associated Receptors TAAR3, TAAR4 and TAAR5 in Primates
Source: PLoS One. 2010 Jun 15;5(6):e11133. doi: 10.1371/journal.pone.0011133 (PMC2886124; doi:10.1371/journal.pone.0011133)
Supplement: Table S2 — Sources of genomic DNA used for TAAR3, TAAR4 and TAAR5 amplification. (0.13 MB PDF) [file pone.0011133.s010.pdf]

species		source
Acinonyx jubatus	cheetah	Dr C. Pitra, IZW, Berlin, Germany
Ailuropoda melanoleuca	giant panda	Dr C. Pitra, IZW, Berlin, Germany
Aotus azarai	Azara's Night Monkey	Dr W. Enard, MPI, Leipzig, Germany
Ateles fusciceps	brown-headed spider monkey	Dr W. Enard, MPI, Leipzig, Germany
Ateles geoffroyi	black-handed spider monkey	Dr W. Enard, MPI, Leipzig, Germany
Bison bonasus	European bison	Dr C. Pitra, IZW, Berlin, Germany
Bos taurus	cattle	Dr J. Chae, University of Chonbuk, Korea
Bradypus tridactylus	pale-throated sloth	Dr M. Hofreiter, MPI, Leipzig, Germany
Callithrix geoffroyi	Geoffroy's marmoset	Dr J. Rhyne, University of Maryland, Baltimore, USA
Callithrix jacchus	common marmoset	Dr T. Haaf, MPI Molecular Genetics Berlin, Germany; Dr W. Enard, MPI, Leipzig, Germany
Camelus bactrianus	bactrian camel	Dr C. Pitra, IZW, Berlin, Germany
Canis familiaris	dog	own source
Canis lupus	gray wolf	Dr C. Pitra, IZW, Berlin, Germany
Cebus apella	tufted capuchin	Dr W. Enard, MPI, Leipzig, Germany
Ceratotherium simum	white rhinoceros	Dr C. Pitra, IZW, Berlin, Germany
Cercopithecus aethiops	African green monkey	ATCC – cell collection
Cervus elaphus	red deer	Dipl. Med. J. Moeller, Erfurt, Germany
Colobus guereza	guereza	Dr W. Enard, MPI, Leipzig, Germany
Dipodomys ordii	Ord's kangaroo rat	
Elephas maximus	asiatic elephant	Dr C. Pitra, IZW, Berlin, Germany
Equus przewalskii	Przewalski horse	Dr C. Pitra, IZW, Berlin, Germany
Erinaceus concolor	eastern European hedgehog	own source
Erythrocebus patas	patas monkey	Dr J. Rhyne, University of Maryland, Baltimore, USA
Galago moholi	South African galago	Dr W. Enard, MPI, Leipzig, Germany
Giraffa camelopardalis	giraffe	Dr C. Pitra, IZW, Berlin, Germany
Gorilla gorilla	Western Gorilla	Dr J. Bullerdiek, University of Bremen; Dr T. Haaf, MPI Molecular Genetics Berlin, Germany; Dr J. Rhyne, University of Maryland, Baltimore, USA; Dr L. Vigilant, MPI, Leipzig, Germany
Hexaprotodon liberiensis	pygmy hippopotamus	Dr C. Pitra, IZW, Berlin, Germany
Hippotragus niger	sable antelope	Dr C. Pitra, IZW, Berlin, Germany
Homo sapiens	human	Dr M. Stoneking, MPI, Leipzig, Germany; D. Sere, MPI, Leipzig, Germany; own source
Hyaena hyaena	striped hyena	Dr C. Pitra, IZW, Berlin, Germany
Hylobates lar	white-handed gibbon	Dipl. Biol. C. Roos, Primate Center Goettingen, Germany; Dr W. Enard, MPI, Leipzig, Germany; Dr J. Bullerdiek, University of Bremen; Dr M. Rocchi, University of Bari, Italy
Lagothrix lagotricha.	common woolly monkey	Dr W. Enard, MPI, Leipzig, Germany
Lemur catta	ring-tailed lemur	Dr J. Rhyne, University of Maryland, Baltimore, USA; Dr W. Enard, MPI, Leipzig, Germany
Loxodonta africana	African savanna elephant	Dr C. Pitra, IZW, Berlin, Germany
Lutra lutra	Eurasian river otter	Dr C. Pitra, IZW, Berlin, Germany
Macaca fascicularis	crab-eating Macaque	Dr W. Enard, MPI, Leipzig, Germany

Macaca mulatta	rhesus monkey	Dr J. Rhyne, University of Maryland, Baltimore, USA; Dr M. Rocchi, University of Bari, Italy; Dr W. Enard, MPI, Leipzig, Germany
Mandrillus sphinx	mandrill	Dr W. Enard, MPI, Leipzig, Germany
Martes foina	beach marten	own source
Meriones meridianus	mid-day jird	Dr C. Pitra, IZW, Berlin, Germany
Meriones unguiculatus	Mongolian gerbil	Research Facility for Experimental Medicine, Berlin, Germany
Mus musculus	house mouse	Dr A. Orth, University of Montpellier, France
Myotis albescens	silver-tipped myotis	Dr C. Pitra, IZW, Berlin, Germany
Nasua nasua	ring-tailed coati	Dr C. Pitra, IZW, Berlin, Germany
Nomascus gabriellae	red-cheeked Gibbon	Dipl. Biol. C. Roos, Primate Center Goettingen, Germany
Nomascus leucogenys	white-cheeked Gibbon	Dipl. Biol. C. Roos, Primate Center Goettingen, Germany
Nyctereutes procyonoides	raccoon dog	Dr C. Pitra, IZW, Berlin, Germany
Nycticebus bengalensis	Bengal slow loris	Dr W. Enard, MPI, Leipzig, Germany
Octodontomys gliroides	Mountain Degu	Dr R. Adkins, University of TN, USA
Ondatra zibethicus	muskrat	Dr Lauenstein, Federal Centre for Agriculture and Forestry, Braunschweig, Germany
Orycteropus afer	aardvark	Dr R. Adkins, University of TN, USA
Ovibos moschatus	muskox	Dr C. Pitra, IZW, Berlin, Germany
Ovis aries	sheep	Dr C. Pitra, IZW, Berlin, Germany
Pan paniscus	bonobo	Dr W. Enard, MPI, Leipzig, Germany; Dr L. Vigilant, MPI, Leipzig, Germany
Pan troglodytes	chimpanzee	Dr T. Haaf, MPI Molecular Genetics Berlin, Germany; Dr W. Enard, MPI, Leipzig, Germany; Dr M. Rocchi, University of Bari, Italy
Panthera leo	lion	Dr C. Pitra, IZW, Berlin, Germany
Panthera onca	jaguar	Dr C. Pitra, IZW, Berlin, Germany
Panthera tigris	tiger	Dr C. Pitra, IZW, Berlin, Germany
Papio hamadryas	hamadryas baboon	Dr M. Rocchi, University of Bari, Italy; Dr W. Enard, MPI, Leipzig, Germany
Phoca sibirica	Baikal seal	Dr C. Pitra, IZW, Berlin, Germany
Pongo pygmaeus	Orangutan	Dr J. Rhyne, University of Maryland, Baltimore, USA; Dr M. Rocchi, University of Bari, Italy; Dr W. Enard, MPI, Leipzig, Germany
Pteropus sp.	flying fox	Dr C. Pitra, IZW, Berlin, Germany
Rattus norvegicus	Norway rat	Research Facility for Experimental Medicine, Berlin, Germany
Rhogeessa io	Thomas's yellow bat	Dr C. Pitra, IZW, Berlin, Germany
Saguinus imperator	emperor tamarin	Dr J. Rhyne, University of Maryland, Baltimore, USA
Saimiri sciureus	common squirrel monkey	Dr W. Enard, MPI, Leipzig, Germany
Symphalangus syndactylus	siamang	Dipl. Biol. C. Roos, Primate Center Goettingen, Germany; Dr T. Haaf, MPI Molecular Genetics Berlin, Germany
Talpa europaea	European mole	own source
Tapirus sp.	tapir	Dr C. Pitra, IZW, Berlin, Germany
Trichechus manatus	Caribbean manatee	Dr C. Pitra, IZW, Berlin, Germany
Uncia uncia	snow leopard	Dr C. Pitra, IZW, Berlin, Germany
Ursus americanus	American black bear	Dr C. Pitra, IZW, Berlin, Germany

Ursus arctos	brown bear	Dr C. Pitra, IZW, Berlin, Germany
Ursus malayanus	Malayan sun bear	Dr C. Pitra, IZW, Berlin, Germany
Ursus maritimus	polar bear	Dr C. Pitra, IZW, Berlin, Germany
Varecia variegata	ruffed lemur	Dr W. Enard, MPI, Leipzig, Germany
Vulpes vulpes	red fox	Dr C. Pitra, IZW, Berlin, Germany
Zalophus californianus	California sea lion	Dr C. Pitra, IZW, Berlin, Germany
