# Supplementary material for: Structural and Functional Evolution of the Trace Amine-Associated Receptors TAAR3, TAAR4 and TAAR5 in Primates
Source: PLoS One. 2010 Jun 15;5(6):e11133. doi: 10.1371/journal.pone.0011133 (PMC2886124; doi:10.1371/journal.pone.0011133)
Supplement: Table S3 — Primers used for TAAR3, TAAR4 and TAAR5 ortholog amplification, sequencing and site-directed introduction of epitope tags. (0.10 MB PDF) [file pone.0011133.s011.pdf]

ID	sequence 5' -> 3'	purpose
OLIGO.123	ATGTGCTTCAGGAAAAAGATTTGC	mouse TAAR3 amplification
OLIGO.124	GATCTAATATACATTCCCGAAGACTTATCC	mouse TAAR3 amplification
OLIGO.153	CCCGACTACGCCGATCTAATATACATTCCCGAAGA	mouse TAAR3 HA-adaptor
OLIGO.154	CATCGTCCTTATAGTCATGTGCTTCAGGAAAAAGA	mouse TAAR3 FLAG-adaptor
OLIGO.188	CCTGGGTTTGTAAGGGTCAA	rat TAAR3 amplification
OLIGO.191	TGTACTCCAGGCAGGTGAAGT	rat TAAR3 amplification
OLIGO.190	CCCGACTACGCCGATCTAATATACATTCCTG	rat TAAR3 HA-adaptor
OLIGO.189	CATCGTCCTTATAGTCATGTGCTTCAGGAAAAAG	rat TAAR3 FLAG-adaptor
OLIGO.178	AGTGACCTGGGGATGTAACG	cow TAAR3 amplification
OLIGO.181	GCTGAAATTTCCTGGGATCA	cow TAAR3 amplification
OLIGO.180	CCCGACTACGCCGATCTAACTTATATTCCTGAAG	cow TAAR3 HA-adaptor
OLIGO.179	CATCGTCCTTATAGTCATGTGCTTCAGGAAACAG	cow TAAR3 FLAG-adaptor
OLIGO.121	ATGTGCTTCAGGAAACAAATTTGCAG	primate TAAR3 amplification
OLIGO.122	GATCTAAYTTAYATTCCCGAMGACCTATC	primate TAAR3 amplification
OLIGO.150	CCCGACTACGCCGATCTAAYTTAYATTCCCGAMGA	primate TAAR3 HA-adaptor
OLIGO.152	CATCGTCCTTATAGTCATGTGCTTCAGGAAACAAA	primate TAAR3 FLAG-adaptor
OLIGO.163	CCTCTCCATGGCAACCA	TAAR3 sequencing
OLIGO.164	CCTGGCTCCATCATGGTT	TAAR3 sequencing
OLIGO.165	GGTACTTCAACTCTACTTGCAACCC	TAAR3 sequencing
OLIGO.174	TGGTTGCCATGGAGAGG	TAAR3 sequencing
OLIGO.175	AACCATGATGGAGCCAGG	TAAR3 sequencing
OLIGO.a	GCCGACTTAGGAATGCAATGTCA	mouse TAAR4 amplification
OLIGO.b	GACCCATCCCTCTTTCCCTTTCT	mouse TAAR4 amplification
OLIGO.55	AATACACCCGACCCCTGGA	mouse TAAR4 re-amplification
OLIGO.158	CCCGACTACGCCAATACACCCGACCCCTGGA	mouse TAAR4 HA-adaptor
OLIGO.171	CCCAAACTTCTACGTGCCTTTCTCCAACAAGACGG GCGTGGTGCGCAATACACCCGACCCCTGGA	mouse TAAR4 rhodopsin-adaptor
OLIGO.c	TTTGGGGAAAACAAGTTCCTGGT	rat TAAR4 amplification
OLIGO.d	TCTGTGAGGTTCAGCACTCATGC	rat TAAR4 amplification
OLIGO.56	AATTCACCTGACCTCTGGTACTCC	rat TAAR4 re-amplification
OLIGO.159	CCCGACTACGCCAATTCACCTGACCTCTGGTACTC	rat TAAR4 HA-adaptor
OLIGO.115	CCCAAACTTCTACGTGCCTTTCTCCAACAAGACGG GCGTGGTGCGCAATTCACCTGACCTCTGGTACTCC	rat TAAR4 rhodopsin-HA-tag
OLIGO.g	CATCGTCCTTATAGTCAGGATGTGCAGGATGC	rat TAAR4 FLAG-adaptor
OLIGO.e	TGCAGACTCTCAACCTCCATTCC	dog TAAR4 amplification
OLIGO.f	AGGCCATGCGACAGAGTTTCTTT	dog TAAR4 amplification
OLIGO.54	AATTCACCTGACATTTACAACCCT	dog TAAR4 re-amplification
OLIGO.157	CCCGACTACGCCAATTCACCTGACATTTACAACCC	dog TAAR4 HA-adaptor
OLIGO.170	CCCAAACTTCTACGTGCCTTTCTCCAACAAGACGG GCGTGGTGCGCAATTCACCTGACATTTACAACCCT	dog TAAR4 rhodopsin-adaptor
OLIGO.36	TCATGATYRGDKCNATAGTRATG	mammal TAAR4 amplifcation
OLIGO.41	ATCATCYTMARKGCYTTDCGAAACCARGGATA	mammal TAAR4 amplifcation
OLIGO.48	TMATAGTAYCCAYRRGACAAAGAYGATG	mammal TAAR4 amplifcation
OLIGO.49	RGGTRGADGAGTCWGRGHGRAAGA	mammal TAAR4 amplifcation

OLIGO.58	AATTCACCTGACCTTTGGAATC	northern treeshrew TAAR4 re-amplification
OLIGO.161	CCCGACTACGCCAATTCACCTGACCTTTGGAATC	northern treeshrew TAAR4 HA-adaptor
OLIGO.173	CCCAAACTTCTACGTGCCTTTCTCCAACAAGACGGGCGTGGTGCGCAATTCACCTGACCTTTGGAATC	northern treeshrew TAAR4 rhodopsin-adaptor
OLIGO.53	AATTTTCCTGACCCTCGGAAT	spider monkey TAAR4 re-amplification
OLIGO.156	CCCGACTACGCCAATTTTCCTGACCCTCGGAAT	spider monkey TAAR4 HA-adaptor
OLIGO.169	CCCAAACTTCTACGTGCCTTTCTCCAACAAGACGGGCGTGGTGCGCAATTTTCCTGACCCTCGGAAT	spider monkey TAAR4 rhodopsin-adaptor
OLIGO.57	AATTTGCCTGACCCTCAGAAC	rhesus monkey, orangutan, siamang TAAR4 re-amplification
OLIGO.160	CCCGACTACGCCAATTTGCCTGACCCTCAGAAC	rhesus monkey, orangutan, siamang TAAR4 HA-adaptor
OLIGO.172	CCCAAACTTCTACGTGCCTTTCTCCAACAAGACGGGCGTGGTGCGCAATTTGCCTGACCCTCAGAAC	rhesus monkey, orangutan, siamang TAAR4 rhodopsin-adaptor
OLIGO.51	AGCAAGGGCAGGAAACAGGCTTARGGTRGADGAGTCWGRGHGRAAGA	reamplification construct TAAR4 C-terminus rhesus monkey
OLIGO.162	CATCGTCCTTATAGTCAGCAAGGGCAGGAAACAGGCTTA	rhesus monkey TAAR4 C-terminus and FLAG adaptor
OLIGO.59	GATCGGGGCTATAGTGATGAC	TAAR4 sequencing
OLIGO.60	GCTGTGTGGTCATGCCYTTCA	TAAR4 sequencing
OLIGO.61	TGGCTACTGTAAAAATRTGTA	TAAR4 sequencing
OLIGO.127	GTCATGGTATAAATCAACAGTCGGTGTCC	mouse TAAR5 amplification
OLIGO.128	AGAGCTGTCCTCCTCCCGGGCTCT	mouse TAAR5 amplification
OLIGO.151	CCCGACTACGCCAGAGCTGTCCTCCTCCCGGGCTC	mouse TAAR5 HA-adaptor
OLIGO.155	CATCGTCCTTATAGTCGTCATGGTATAAATCAACA	mouse TAAR5 FLAG-adaptor
OLIGO.192	CACCGTCTTCCTTCCTGCTA	rat TAAR5 amplification
OLIGO.195	AAAGGTGACCAGTTCCCAGA	rat TAAR5 amplification
OLIGO.194	CCCGACTACGCCAGAGCTGTCCTCCTCCCC	rat TAAR5 HA-adaptor
OLIGO.193	CATCGTCCTTATAGTCGTCATGGAACAGATCAGCA	rat TAAR5 FLAG-adaptor
OLIGO.182	CAATGGACATGAGTTTGAGCA	cow TAAR5 amplification
OLIGO.185	CAAATCTTCCTTTGGTTCTTCC	cow TAAR5 amplification
OLIGO.184	CCCGACTACGCCAGTGTGGTCCTCAACCA	cow TAAR5 HA-adaptor
OLIGO.183	CATCGTCCTTATAGTCATCTTGGTACAAATCAATT	cow TAAR5 FLAG-adaptor
OLIGO.196	ACCTACCCCTTTCCTGCATT	dog TAAR5 amplification
OLIGO.197	GGTGGTCAGTTCCCAGAGAG	dog TAAR5 amplification
OLIGO.187	CCCGACTACGCCGACACTGTCCTCAGCCAAG	dog TAAR5 HA-adaptor
OLIGO.186	CATCGTCCTTATAGTCTTCTTGGTACAAATCAATA	dog TAAR5 FLAG-adaptor
OLIGO.125	TTCTTGGTACAAATCAACAGTGC	primate TAAR5 amplification
OLIGO.126	AGAGCTGTCTTYATCCAAGGTG	primate TAAR5 amplification
OLIGO.148	CCCGACTACGCCAGAGCTGTCTTYATCCAAGGTG	primate TAAR5 HA-adaptor
OLIGO.149	CATCGTCCTTATAGTCTTCTTGGTACAAATCAACA	primate TAAR5 FLAG-adaptor
OLIGO.166	GTGGAGAGCTGCTGGTTCTT	TAAR5 sequencing
OLIGO.167	ATGCCTTGTGTGGGCAG	TAAR5 sequencing
OLIGO.168	GTCTTTGACATCTTTATCTGGTTTGC	TAAR5 sequencing

OLIGO.176	AAGAACCAGCAGCTCTCCAC	TAAR5 sequencing
OLIGO.177	GCAAACCAGATAAAGATGTCAAAGAC	TAAR5 sequencing
OLIGO.0	CGCGAATTCCCCACCATGTACCCCTACGACGTCCC	HA uni EcoRI+Coseq
OLIGO.113	CTCGAATTCCCCACCATGTACCCCTACGACGTCCC CGACTACGCCAACGGGACCGAGGGCCCAAACTTC TACGTGCCTTTC	HA-Rhod-S-uni
OLIGO.1	CGCCGCACTAGTTCACTTATCGTCATCGTCCTTAT	FLAG uni SpeI
