# Supplementary material for: Structural and Functional Evolution of the Trace Amine-Associated Receptors TAAR3, TAAR4 and TAAR5 in Primates
Source: PLoS One. 2010 Jun 15;5(6):e11133. doi: 10.1371/journal.pone.0011133 (PMC2886124; doi:10.1371/journal.pone.0011133)
Supplement: Table S4 — Phylogenetic trees in NEWICK notation. (0.10 MB PDF) [file pone.0011133.s012.pdf]

primate TAAR3 tree (ORF and pseudogene lineages)*
((((((((HsapiTAAR3,PtrogTAAR3),GgoriTAAR3),PpygmTAAR3),(HlarTAAR3,SsyndTAAR3)),(((MmulaTAAR3,MfascTAAR3),PhamaTAAR3),EpataTAAR3)),((((CjaccTAAR3,CgeofTAAR3),SimpeTAAR3),(SsciuTAAR3,AazarTAAR3)),((AfuscTAAR3,AgeofTAAR3),LlagoTAAR3))),TsyriTAAR3),((MmuriTAAR3,LcattTAAR3),OgarnTAAR3));
primate TAAR3 tree (ORF lineages)*
((((SsyndTAAR3,(((MmulaTAAR3,MfascTAAR3),PhamaTAAR3),EpaTAARTAAR3)),((SsciuTAAR3,AazarTAAR3),(AfuscTAAR3,AgeofTAAR3))),TsyriTAAR3),((MmuriTAAR3,LcattTAAR3),OgarnTAAR3));
primate TAAR3 common tree[+]
((((((HsapiTAAR3:0.00547266,GgoriTAAR3:0.01045161)0.47:0.00096179,PtrogTAAR3:0.00625624)0.96:0.00783630,(HlarTAAR3:0.02187465,SsyndTAAR3:0.00890499)0.99:0.00894758)0.27:0.00008673,PpygmTAAR3:0.01001994)0.65:0.00236339,((PhamaTAAR3:0.00139957,MmulaTAAR3:0.00145430)0.78:0.00136628,EpataTAAR3:0.00436069)1.00:0.01163620)1.00:0.01429488,(((((CjaccTAAR3:0.00620937,CgeofTAAR3:0.00674104)0.98:0.00636748,SimpeTAAR3:0.02302708)0.51:0.00104933,SsciuTAAR3:0.00758429)0.39:0.00086236,((AfuscTAAR3:0.00428642,AgeofTAAR3:0.00143162)0.98:0.00362473,LlagoTAAR3:0.01377462)0.93:0.00527155)0.42:0.00102703,AazarTAAR3:0.00796530)1.00:0.01302818,(((MmuriTAAR3:0.01641716,LcattTAAR3:0.01568312)1.00:0.01989724,OgarnTAAR3:0.06041266)0.47:0.00204571,TsyriTAAR3:0.06468666)1.00:0.04149377);
primate TAAR3 common tree (only ω-ratios that were included in correlation analyses are indicated)[#]
((((((((HsapiTAAR3,PtrogTAAR3'#1.6695'),GgoriTAAR3'#2.5189')'#0.2678',PpygmTAAR3'#0.7906'),(HlarTAAR3,SsyndTAAR3)'#0.1244'),((MmulaTAAR3,PhamaTAAR3),EpataTAAR3)'#0.1240')'#0.3897',((((CjaccTAAR3'#0.4034',CgeofTAAR3'#0.0995')'#1.5660',SimpeTAAR3'#1.2141'),(SsciuTAAR3'#0.2995',AazarTAAR3'#0.2006')),((AfuscTAAR3,AgeofTAAR3)'#0.4070',LlagoTAAR3'#0.5941')'#0.5966')'#0.2126')'#0.1366',TsyriTAAR3'#0.1811'),((MmuriTAAR3'#0.0389',LcattTAAR3),OgarnTAAR3'#0.0485'));
primate TAAR4 tree (ORF and pseudogene lineages)*
((((((((HsapiTAAR4,(PtrogTAAR4,PpaniTAAR4)),GgoriTAAR4),PpygmTAAR4),((NleucTAAR4,NgabrTAAR4),(HlarTAAR4,SsyndTAAR4))),((((PhamaTAAR4,MsphiTAAR4),MmulaTAAR4),EpataTAAR4),CguerTAAR4)),((((CapelTAAR4,SsciuTAAR4),AazarTAAR4),((CjaccTAAR4,CgeofTAAR4),SimpeTAAR4)),((AfuscTAAR4,AgeofTAAR4),LlagoTAAR4))),TsyriTAAR4),(((VvariTAAR4,LcattTAAR4),MmuriTAAR4),(OgarnTAAR4,NbengTAAR4)));
primate TAAR4 tree (ORF lineages)*
(((((PpygmTAAR4,SsyndTAAR4),((((PhamaTAAR4,MsphiTAAR4),MmulaTAAR4),EpataTAAR4),CguerTAAR4)),(((CapelTAAR4,SsciuTAAR4),AazarTAAR4),((AfuscTAAR4,AgeofTAAR4),LlagoTAAR4))),TsyriTAAR4),(((VvariTAAR4,LcattTAAR4),MmuriTAAR4),(OgarnTAAR4,NbengTAAR4)));
primate TAAR4 common tree[+]
(((((((HsapiTAAR4:0.00668468,PtrogTAAR4:0.00873595)0.83:0.00217870,GgoriTAAR4:0.01480982)0.98:0.00526092,PpygmTAAR4:0.00889845)0.46:0.00180100,(HlarTAAR4:0.01046556,SsyndTAAR4:0.01069514)1.00:0.01152589)0.69:0.00194117,((MmulaTAAR4:0.00434124,PhamaTAAR4:0.00122062)1.00:0.00726298,EpataTAAR4:0.00965273)0.94:0.00582866)1.00:0.01101951,(SimpeTAAR4:0.01540514,((CjaccTAAR4:0.00417831,CgeofTAAR4:0.00558953)1.00:0.01527163,((AazarTAAR4:0.01356850,SsciuTAAR4:0.02346234)0.11:0.00029235,((AgeofTAAR4:0.00146775,AfuscTAAR4:-0.00008199)1.00:0.01170322,LlagoTAAR4:0.01082904)0.62:0.00219772)0.28:0.00215837)0.49:0.00208467)0.99:0.00955305)1.00:0.02982009,((LcattTAAR4:0.01510832,MmuriTAAR4:0.02185054)0.98:0.01347387,OgarnTAAR4:0.04845674)1.00:0.01773647,TsyriTAAR4:0.08223906);
primate TAAR4 common tree (only ω-ratios that were included in correlation analyses are indicated)[#]
((((((((HsapiTAAR4,PtrogTAAR4'#2.0362'),GgoriTAAR4'#0.9245')'#0.3730',PpygmTAAR4'#0.1966'),(HlarTAAR4,SsyndTAAR4)'#0.2139'),((MmulaTAAR4,PhamaTAAR4),EpataTAAR4)'#0.7212')'#0.2278',((((CjaccTAAR4'#0.1953',CgeofTAAR4'#1.1579')'#3.5336',SimpeTAAR4'#0.2788'),(SsciuTAAR4'#0.3273',AazarTAAR4'#0.5095')),((AfuscTAAR4,AgeofTAAR4)'#1.2227',LlagoTAAR4'#0.3839')'#0.3984')'#0.1597')'#0.1305',TsyriTAAR4'#0.2513'),((MmuriTAAR4'#0.0855',LcattTAAR4),OgarnTAAR4'#0.1441'));
primate TAAR5 tree (ORF and pseudogene lineages)*
((((((((HsapiTAAR5,PtrogTAAR5),GgoriTAAR5),PpygmTAAR5),((NleucTAAR5,NgabrTAAR5),(HlarTAAR5,SsyndTAAR5))),((MmulaTAAR5,PhamaTAAR5),EpataTAAR5)),(((SsciuTAAR5,AazarTAAR5),((CjaccTAAR5,CgeofTAAR5),SimpeTAAR5)),((AfuscTAAR5,AgeofTAAR5),LlagoTAAR5))),TsyriTAAR5),((MmuriTAAR5,LcattTAAR5),OgarnTAAR5));
primate TAAR5 tree (ORF lineages)*
(((((((HsapiTAAR5,PtrogTAAR5),GgoriTAAR5),PpygmTAAR5),(HlarTAAR5,SsyndTAAR5)),((MmulaTAAR5,PhamaTAAR5),EpataTAAR5)),(((SsciuTAAR5,AazarTAAR5),((CjaccTAAR5,CgeofTAAR5),SimpeTAAR5)),((AfuscTAAR5,AgeofTAAR5),LlagoTAAR5))),((MmuriTAAR5,LcattTAAR5),OgarnTAAR5));
primate TAAR5 common tree[+]
(((((((HsapiTAAR5:0.00230778,PtrogTAAR5:0.00703937)0.37:0.00001357,GgoriTAAR5:0.00464382)0.99:0.00405948,PpygmTAAR5:0.01599822)0.40:0.00037594,(HlarTAAR5:0.00679578,SsyndTAAR5:0.00134998)0.94:0.00374697)0.52:0.00092352,(MmulaTAAR5:0.00445966,(PhamaTAAR5:0.00068329,EpataTAAR5:0.00396189)0.75:0.00136861)1.00:0.01066631)0.84:0.00458882,(((((CjaccTAAR5:0.00358936,CgeofTAAR5:0.00339535)1.00:0.01234796,SimpeTAAR5:0.00412750)0.95:0.00330378,AazarTAAR5:0.01088414)0.58:0.00116702,SsciuTAAR5:0.00938892)0.78:0.00142025,((AfuscTAAR5

:0.00756803,AgeofTAAR5:0.00176572)0.99:0.00682722,LlagoTAAR5:0.00489860)1.00:0.00954950)0.95:0.00617870)1.00:0.02378173,((LcattTAAR5:0.03287626,MmuriTAAR5:0.02152262)1.00:0.01728956,OgarnTAAR5:0.03676877)0.97:0.01034600,TsyriTAAR5:0.09951675);
primate TAAR5 common tree (only ω-ratios that were included in correlation analyses are indicated)[#]
((((((((HsapiTAAR5,PtrogTAAR5'#0.3558'),GgoriTAAR5'#1.0759')'#0.7099',PpygmTAAR5'#0.1936'),(HlarTAAR5,SsyndTAAR5)'#0.1753'),((MmulaTAAR5,PhamaTAAR5),EpataTAAR5)'#0.1295')'#0.1189',((((CjaccTAAR5'#0.7402',CgeofTAAR5'#0.7258')'#0.2928',SimpeTAAR5'#0.1839'),(SsciuTAAR5'#0.0477',AazarTAAR5'#0.2538')),((AfuscTAAR5,AgeofTAAR5)'#0.1945',LlagoTAAR5'#0.1018')'#0.4432')'#0.1338')'#0.3119',TsyriTAAR5'#0.2475'),((MmuriTAAR5'#0.1164',LcattTAAR5),OgarnTAAR5'#0.1164'));
TAAR-mammal-tree*
(MdomeTAAR,((EtelfTAAR,(PcapeTAAR,LafriTAAR)),(DnoveTAAR,((SaranTAAR,(MluciTAAR,((BtaurTAAR,SscroTAAR),EcabaTAAR))),(OcuniTAAR,(CporcTAAR,(MmuscTAAR,RnorvTAAR)))))));

*Phylogentic trees that were used for PAML analyses shown in Table S6; [+]The evolutionary history of 21 primates was inferred using the Neighbor-Joining method [1]. The bootstrap consensus tree inferred from 1,000 replicates is taken to represent the evolutionary history of the taxa analyzed [6]. The percentage of replicate trees in which the associated taxa clustered together in the bootstrap test (1,000 replicates) are indicated next to the branches [6]. The evolutionary distances were computed using the Maximum Composite Likelihood model [2]. The tree is drawn to scale, with branch length corresponding to nucleotide substitutions per site. All codon positions were included, all postions containing gaps and missing data were eliminated from the dataset. There were a total of 702 nucleotides in the final TAAR3 dataset, 723 nucleotides in the final TAAR4 dataset and 864 nucleotides in the final TAAR5 dataset. Phylogenetic analyses were conducted in MEGA4 [3]. [#]ω-ratios were determined in PAML using a „free-ratios" model. ω-ratios are not indicated for branches which showed d_S=0 or d_N=0 in one of the 3 TAAR orthologs because they were not included in Spearman rank correlation analyses.
Aazar: Azara's night monkey, Afusc: brown-headed spidermonkey, Ageof: black-handed spider monkey, Btaur: cattle, Capel: tufted capuchin, Cgeof: Geoffroy's marmoset, Cguer: guereza, Cjacc: common marmoset, Cporc: domestic guinea pig, Dnove: nine-banded armadillo, Ecaba: horse, Epata: patas monkey, Etelf: lesser hedgehog tenrec, Ggori: gorilla, Hlar: white-handed gibbon, Hsapi: human, Lafri: African savanna elephant, Lcatt: ring-tailed lemur, Llago: common woolly monkey, Mdome: gray short-tailed opossum, Mfasc: crab-eating macaque, Mluci: little brown bat, Mmula: rhesus monkey, Mmuri: gray mouse lemur, Mmusc: house mouse, Msphi: mandrill, Nbeng: Bengal slow loris, Ngabr: yellow-cheeked gibbon, Nleuc: white-cheeked gibbon, Ocuni: European rabbit, Ogarn: small-eared galago, Pcape: cape rock hyrax, Phama: hamadryas baboon, Ppani: bonobo, Ppygm: orangutan, Ptrog: chimpanzee, Rnorv: Norway rat, Saran: European shrew, Simpe: emperor tamarin, Ssciu: common squirrel monkey, Sscrof: boar, Ssynd: siamang, Tsyri: Philippine tarsier, Vvari: ruffed lemur
