# Supplementary material for: Structural and Functional Evolution of the Trace Amine-Associated Receptors TAAR3, TAAR4 and TAAR5 in Primates
Source: PLoS One. 2010 Jun 15;5(6):e11133. doi: 10.1371/journal.pone.0011133 (PMC2886124; doi:10.1371/journal.pone.0011133)
Supplement: Table S5 — Structural comparison of mammalian GPCR orthologs. The amino acid sequence information of 8 full-length orthologs (Bos taurus, Cavia porcellus, Echinops telfairi, Macaca mulatta, Mus musculus, Oryctolagus cuniculus, Rattus norvegicus, Sus scrofa) of each receptor was used to determine the structural conservation between mammalian orthologs (given as % aa identity determined by ClustalW implemented in MegAlign of Lasergene 7.1.) are shown. Data are given as mean±S.D. ADRB1, beta-1-adrenergic receptor; ADRB2, beta-2-adrenergic receptor; MC4R, melancortin receptor 4; V2R, vasopressin type 2 receptor. (0.05 MB PDF) [file pone.0011133.s013.pdf]

receptor	conservation between orthologs (% aa identity)
Rhodopsin	94.2±1.3
ADRB1	92.2±2.8
ADRB2	87.3±2.8
MC4R	94.0±2.0
V2R	85.4±3.1
TAAR1	78.9±5.9
TAAR2	88.9±2.3
TAAR3	87.2±2.7
TAAR4	83.5±3.2
TAAR5	85.6±2.6
TAAR6	84.1±3.3
