# Supplementary material for: Structural and Functional Evolution of the Trace Amine-Associated Receptors TAAR3, TAAR4 and TAAR5 in Primates
Source: PLoS One. 2010 Jun 15;5(6):e11133. doi: 10.1371/journal.pone.0011133 (PMC2886124; doi:10.1371/journal.pone.0011133)
Supplement: Table S6 — Maximum likelihood estimates of dN/dS ratios (ω) for primate and 14 non-primate mammalian TAAR3, TAAR4 and TAAR5 under different models using PAML. (0.19 MB PDF) [file pone.0011133.s014.pdf]

**models**	p	ℓ	κ	**estimates of parameters**
**TAAR3**				
primate ORF and pseudogene lineages				
Branch-specific models:				
A: Free ratio	85	-2584.43	4.23	
B: One ratio: ω_0	44	-2639.40	4.14	$\omega_0 = 0.218$
C: Two ratios: ω_0, ω_ψ	45	-2614.84	4.18	$\omega_0 = 0.128$, $\omega_\psi = 0.878$
D: Two ratios: ω_0, $\omega_\psi = 1$	44	-2614.98	4.21	$\omega_0 = 0.128$, $\omega_\psi = 1$
E: Two ratios: $\omega_0 = 1$, ω_ψ	44	-2721.52	5.69	$\omega_0 = 1$, $\omega_\psi = 0.920$
F: Three ratios: ω_0, $\omega_{\psi1}$, $\omega_{\psi2}$	46	-2612.82	4.18	$\omega_0 = 0.128$, $\omega_{\psi1} = 2.915$, $\omega_{\psi2} = 0.701$
G: Three ratios: ω_0, $\omega_{\psi1} = 1$, $\omega_{\psi2}$	45	-2614.10	4.14	$\omega_0 = 0.128$, $\omega_{\psi1} = 1$, $\omega_{\psi2} = 0.699$
H: Three ratios: ω_0, $\omega_{\psi1}$, $\omega_{\psi2} = 1$	45	-2613.69	4.24	$\omega_0 = 0.129$, $\omega_{\psi1} = 2.924$, $\omega_{\psi2} = 1$
primate ORF lineages				
Branch-specific models:				
Free ratio	49	-2036.22	4.39	
One ratio: ω_0	26	-2054.05	4.35	$\omega_0 = 0.122$
Site-specific models:				
M1a (neutral)	27	-2047.31	4.46	$p_0 = 0.901$, $p_1 = 0.099$
				$\omega_0 = 0.061$, $\omega_1 = 1$
M2a (selection)	29	-2047.31	4.47	$p_0 = 0.901$, $p_1 = 0.079$, $p_2 = 0.021$
				$\omega_0 = 0.061$, $\omega_1 = 1$, $\omega_2 = 1$
M3 (discrete)	30	-2044.48	4.29	$p_0 = 0.398$, $p_1 = 0.269$, $p_2 = 0.333$
				$\omega_0 = 0.000$, $\omega_1 = 0.000$, $\omega_2 = 0.397$
M7 (beta)	27	-2045.03	4.33	$p = 0.211$, $q = 1.327$
M8 (beta& ω)	29	-2045.03	4.33	$p_0 = 0.999$, $p = 0.211$, $q = 1.327$,
				($p_1 = 0.00001$), $\omega = 1.000$
14 non-primate mammalians				
Branch-specific models:				
Free ratio	53	-5943.61	2.93	
One ratio: ω_0	28	-5974.48	2.87	$\omega_0 = 0.106$
Site-specific models				
M1a (neutral)	29	-5884.20	3.08	$p_0 = 0.881$, $p_1 = 0.119$
				$\omega_0 = 0.058$, $\omega_1 = 1$
M2a (selection)	31	-5884.20	3.08	$p_0 = 0.881$, $p_1 = 0.116$, $p_2 = 0.004$
				$\omega_0 = 0.058$, $\omega_1 = 1$, $\omega_2 = 1$
M3 (discrete)	32	-5851.43	2.91	$p_0 = 0.532$, $p_1 = 0.323$, $p_2 = 0.145$
				$\omega_0 = 0.007$, $\omega_1 = 0.125$, $\omega_2 = 0.541$
M7 (beta)	29	-5851.94	2.90	$p = 0.260$, $q = 1.795$
M8 (beta& ω)	31	-5851.94	2.90	$p_0 = 1$, $p = 0.260$, $q = 1.795$
				($p_1 = 0$), $\omega = 7.132$
**TAAR4**				
primate ORF and pseudogene lineages				
Branch-specific models:				
A: Free ratio	113	-3196.81	3.91	
B: One ratio: ω_0	58	-3248.05	3.86	$\omega_0 = 0.338$
C: Two ratios: ω_0, ω_ψ	59	-3238.77	3.88	$\omega_0 = 0.274$, $\omega_\psi = 0.884$
D: Two ratios: ω_0, $\omega_\psi = 1$	58	-3238.88	3.89	$\omega_0 = 0.274$, $\omega_\psi = 1$
E: Two ratios: $\omega_0 = 1$, ω_ψ	58	-3300.21	4.64	$\omega_0 = 1$, $\omega_\psi = 0.903$
F: Three ratios: ω_0, $\omega_{\psi1}$, $\omega_{\psi2}$	60	-3238.68	3.88	$\omega_0 = 0.274$, $\omega_{\psi1} = 0.787$, $\omega_{\psi2} = 0.982$

G: Three ratios: ω_0, $\omega_{\psi 1} = 1$, $\omega_{\psi 2}$	59	-3238.88	3.89	$\omega_0 = 0.274$, $\omega_{\psi 1} = 1$, $\omega_{\psi 2} = 0.982$
H: Three ratios: ω_0, $\omega_{\psi 1}$, $\omega_{\psi 2} = 1$	59	-3238.68	3.88	$\omega_0 = 0.274$, $\omega_{\psi 1} = 0.787$, $\omega_{\psi 2} = 1$
primate ORF lineages				
Branch-specific models:				
Free ratio	73	-2679.78	4.02	
One ratio: ω_0	38	-2706.23	3.98	$\omega_0 = 0.272$
Two ratios: ω_0, ω_{cerc}	39	-2699.06	3.99	$\omega_0 = 0.247$, $\omega_{cerc} = 2.590$
Two ratios: ω_0, $\omega_{cerc} = 1$	38	-2700.03	3.97	$\omega_0 = 0.246$, $\omega_{cerc} = 1$
Site-specific models:				
M1a (neutral)	39	-2693.26	3.98	$p_0 = 0.825$, $p_1 = 0.175$<br>$\omega_0 = 0.148$, $\omega_1 = 1$
M2a (selection)	41	-2693.26	3.98	$p_0 = 0.825$, $p_1 = 0.110$, $p_2 = 0.066$<br>$\omega_0 = 0.148$, $\omega_1 = 1$, $\omega_2 = 1$
M3 (discrete)	42	-2692.47	3.98	$p_0 = 0.290$, $p_1 = 0.650$, $p_2 = 0.059$<br>$\omega_0 = 0.000$, $\omega_1 = 0.312$, $\omega_2 = 1.616$
M7 (beta)	39	-2693.63	4.00	$p = 0.471$, $q = 1.159$
M8 (beta& ω)	41	-2692.65	3.98	$p_0 = 0.953$, $p = 0.879$, $q = 2.884$<br>($p_1 = 0.047$), $\omega = 1.671$
Branch-site models:				
Model A (foreground branch: cerc)	41	-2688.97	4.01	$p_0 = 0.388$, $\omega_{0(othprim)} = 0.153$,<br>$\omega_{0(cerc)} = 0.153$<br>$p_1 = 0.061$, $\omega_{1(othprim)} = 1$, $\omega_{1(cerc)} = 1$<br>$p_{2a} = 0.476$, $\omega_{2a(othprim)} = 0.153$,<br>$\omega_{2a(cerc)} = 3.722$<br>$p_{2b} = 0.075$; $\omega_{2b(othprim)} = 1$,<br>$\omega_{2b(cerc)} = 3.722$<br>Pos16, Pos37, Pos55, Pos110, Pos 113, Pos 126, Pos129, Pos158, Pos189, Pos193, Pos227, Pos236 (P>0.8)
Model A'(foreground branch: cerc, $\omega_2 = 1$)	40	-2690.06	3.98	$p_0 = 0.000$, $\omega_{0(othprim)} = 0.155$,<br>$\omega_{0(cerc)} = 0.155$<br>$p_1 = 0.000$, $\omega_{1(othprim)} = 1$, $\omega_{1(cerc)} = 1$<br>$p_{2a} = 0.870$, $\omega_{2a(othprim)} = 0.155$,<br>$\omega_{2a(cerc)} = 1$<br>$p_{2b} = 0.130$; $\omega_{2b(othprim)} = 1$, $\omega_{2b(cerc)} = 1$
14 non-primate mammalians				
Branch-specific models:				
Free ratio	53	-6627.75	3.10	
One ratio: ω_0	28	-6667.08	3.05	$\omega_0 = 0.176$
Two ratio ω_0, ω_{glires}	29	-6665.12	3.06	$\omega_0 = 0.172$, $\omega_{glires} = 1.209$
Two ratio ω_0, $\omega_{glires}=1$	28	-6665.13	3.06	$\omega_0 = 0.172$, $\omega_{glires} = 1$
Site-specific models				
M1a (neutral)	29	-6528.53	3.23	$p_0 = 0.792$, $p_1 = 0.208$<br>$\omega_0 = 0.079$, $\omega_1 = 1$
M2a (selection)	31	-6528.53	3.23	$p_0 = 0.792$, $p_1 = 0.151$, $p_2 = 0.057$<br>$\omega_0 = 0.079$, $\omega_1 = 1$, $\omega_2 = 1$
M3 (discrete)	32	-6507.41	3.08	$p_0 = 0.616$, $p_1 = 0.282$, $p_2 = 0.102$<br>$\omega_0 = 0.035$, $\omega_1 = 0.334$, $\omega_2 = 0.958$
M7 (beta)	29	-6510.06	3.06	$p = 0.320$, $q = 1.203$
M8 (beta& ω)	31	-6507.87	3.08	$p_0 = 0.915$, $p = 0.445$, $q = 2.645$<br>($p_1 = 0.085$), $\omega = 1$<br>Pos12, Pos38, Pos235, Pos238, Pos251, (P>0.7)

Branch-site models:				
Model A (foreground branch: *glires*)	31	-6526.16	3.23	$p_0 = 0.783$, $\omega_{0(othmam)} = 0.079$, $\omega_{0(glires)} = 0.079$;<br>$p_1 = 0.198$, $\omega_{1(othmam)} = 1$, $\omega_{1(glires)} = 1$<br>$p_{2a} = 0.015$, $\omega_{2a(othmam)} = 0.079$, $\omega_{2a(glires)} = 42.11$<br>$p_{2b} = 0.004$; $\omega_{2b(othmam)} = 1$, $\omega_{2b(glires)} = 42.11$<br>Pos 32, Pos 332 (P>0.7)
Model A' (foreground branch: *glires*, $\omega_2 = 1$)	30	-6528.04	3.23	$p_0 = 0.677$, $\omega_{0(othmam)} = 0.079$, $\omega_{0(glires)} = 0.079$<br>$p_1 = 0.173$, $\omega_{1(othmam)} = 1$, $\omega_{1(glires)} = 1$<br>$p_{2a} = 0.120$, $\omega_{2a(othmam)} = 0.079$, $\omega_{2a(glires)} = 1$<br>$p_{2b} = 0.031$; $\omega_{2b(othmam)} = 1$, $\omega_{2b(glires)} = 1$
**TAAR5**				
primate ORF and pseudogene lineages				
Branch-specific models:				
A: Free ratio	89	-3115.27	3.34	
B: One ratio: ω_0	46	-3133.45	3.34	$\omega_0 = 0.236$
C: Two ratios: ω_0, ω_ψ	47	-3132.24	3.33	$\omega_0 = 0.213$, $\omega_\psi = 0.319$
D: Two ratios: ω_0, ω_ψ= 1	46	-3144.46	3.49	$\omega_0 = 0.213$, $\omega_\psi = 1$
E: Two ratios: $\omega_0 = 1$, ω_ψ	46	-3204.57	4.14	$\omega_0 = 1$, $\omega_\psi = 0.323$
F: Three ratios: ω_0, $\omega_{\psi1}$, $\omega_{\psi2}$	48	-3129.08	3.33	$\omega_0 = 0.213$, $\omega_{\psi1} = \infty$, $\omega_{\psi2} = 0.285$
G: Three ratios: ω_0, $\omega_{\psi1} = 1$, $\omega_{\psi2}$	47	-3130.32	3.32	$\omega_0 = 0.213$, $\omega_{\psi1} = 1$, $\omega_{\psi2} = 0.285$
H: Three ratios: ω_0, $\omega_{\psi1}$, $\omega_{\psi2} = 1$	47	-3143.21	3.50	$\omega_0 = 0.213$, $\omega_{\psi1} = \infty$, $\omega_{\psi2} = 1$
primate ORF lineages				
Branch-specific models:				
Free ratio	77	-2684.71	3.35	
One ratio: ω_0	40	-2696.73	3.35	$\omega_0 = 0.213$
Two ratios: ω_0, ω_{hcg}	41	-2694.42	3.37	$\omega_0 = 0.204$, $\omega_{hcg} = 0.537$
Two ratios: ω_0, ω_{hcg}=1	40	-2695.07	3.39	$\omega_0 = 0.205$, $\omega_{hcg} = 1$
Two ratios: ω_0, ω_{rp}	41	-2695.50	3.37	$\omega_0 = 0.213$, $\omega_{rp} = 0.543$
Two ratios: ω_0, ω_{rp}=1	40	-2695.71	3.37	$\omega_0 = 0.213$, $\omega_{rp} = 1$
Site-specific models:				
M1a (neutral)	41	-2692.46	3.41	$p_0 = 0.905$, $p_1 = 0.095$<br>$\omega_0 = 0.151$, $\omega_1 = 1$
M2a (selection)	43	-2692.46	3.41	$p_0 = 0.905$, $p_1 = 0.063$, $p_2 = 0.032$<br>$\omega_0 = 0.151$, $\omega_1 = 1$, $\omega_2 = 1$
M3 (discrete)	44	-2691.44	3.41	$p_0 = 0.353$, $p_1 = 0.636$, $p_2 = 0.010$<br>$\omega_0 = 0.000$, $\omega_1 = 0.321$, $\omega_2 = 2.520$
M7 (beta)	41	-2691.96	3.40	$p = 0.616$, $q = 2.082$
M8 (beta& ω)	43	-2691.70	3.41	$p_0 = 0.992$, $p = 0.837$, $q = 3.068$<br>($p_1 = 0.008$), $\omega = 2.496$
14 non-primate mammalians				
Branch-specific models:				
Free ratio	53	-6044.80	3.45	
One ratio: ω_0	28	-6087.09	3.34	$\omega_0 = 0.143$
Site-specific models:				
M1a (neutral)	29	-5959.23	3.64	$p_0 = 0.814$, $p_1 = 0.185$<br>$\omega_0 = 0.061$, $\omega_1 = 1$

M2a (selection)	31	-5954.77	3.68	$p_0 = 0.813$, $p_1 = 0.183$, $p_2 = 0.004$<br>$\omega_0 = 0.061$, $\omega_1 = 1$, $\omega_2 = 4.99$<br>Pos5*, Pos41 (P>0.7)
M3 (discrete)	32	-5923.02	3.43	$p_0 = 0.683$, $p_1 = 0.296$, $p_2 = 0.021$<br>$\omega_0 = 0.025$, $\omega_1 = 0.407$, $\omega_2 = 2.099$
M7 (beta)	29	-5933.52	3.43	$p = 0.239$, $q = 1.097$
M8 (beta& ω)	31	-5924.12	3.43	$p_0 = 0.988$, $p = 0.282$, $q = 1.532$<br>($p_1 = 0.012$), $\omega = 2.769$<br>Pos5**, Pos41*, Pos12, Pos175<br>(P>0.8)

p, number of free parameters; ℓ, Log likelihood; κ, transition to transversion rate ratio; ω, d_N/d_S ratio; ω_0, indicates ω of all other branches (the ones that are not specifically labeled in a model); $\omega_{\psi 1}$, ω of branches being definitely under neutral evolution because of deleterious mutation (pseudogenization) in ancestor branch; $\omega_{\psi 2}$, ω of branches along which inactivating pseudogenization event occurred (see Figure 1-3 ×); ω_{ψ}, ω of all pseudogene branches (ψ_1 plus ψ_2); ω_{cerc}, ω of *Cercopithecinae* (see Figure 2 for labeling); ω_{hcg}, ω of human, chimp and gorilla; ω_{rp}, ω rhesus monkey and hamadryas baboon; $\omega_{othprim}$, ω of all other primates except species labeled as foreground branches in respective branch-site test of positive selection; ω_{glires}, ω of *Glires* (see Figure S7B for labeling); ω_{othmam}, ω of all other mammals except *Glires* labeled as background branches in branch-site test of positive selection; ∞, ω estimated to be infinite generated by d_S=0 (absence of synonymous changes); * indicates P>0.95; ** indicates P>0.99)
