# Supplementary material for: Structural and Functional Evolution of the Trace Amine-Associated Receptors TAAR3, TAAR4 and TAAR5 in Primates
Source: PLoS One. 2010 Jun 15;5(6):e11133. doi: 10.1371/journal.pone.0011133 (PMC2886124; doi:10.1371/journal.pone.0011133)
Supplement: Table S7 — Likelihood ratio test (LRT) statistics for testing site-specific models within primate ORF lineages and 14 non-primate mammalians. (0.15 MB PDF) [file pone.0011133.s015.pdf]

models compared		M0 (one ratio) vs. M3 (discrete)	M1a (neutral) vs. M2a (selection)	M7 (beta) vs. M8 (beta&ω)
**TAAR3**				
primate ORF lineages				
	df	4	2	2
	2Δℓ	19.13	0.00	0.00
	P-value	0.0007	1	1
14 non-primate mammalians				
	df	4	2	2
	2Δℓ	246.12	0.00	0.01
	P-value	< 0.0001	1	0.9950
**TAAR4**				
primate ORF lineages				
	df	4	2	2
	2Δℓ	27.52	0.00	1.96
	P-value	< 0.0001	1	0.3753
14 non-primate mammalians				
	df	4	2	2
	2Δℓ	319.34	0.00	4.40
	P-value	< 0.0001	1	0.1108
**TAAR5**				
primate ORF lineages				
	df	4	2	2
	2Δℓ	10.58	0.00	0.52
	P-value	0.0317	1	0.7711
14 non-primate mammalians				
	df	4	2	2
	2Δℓ	328.13	8.94	18.82
	P-value	< 0.0001	0.0114	< 0.0001

LRT tests were performed between nested models. df, degrees of freedom, equal to differences in the number of parameters between models; 2Δℓ, likelihood ratio statistic is distributed approximately as χ^2
